# Decoupling Protein Concentration and Aggregate Content Using Diffusion and Water NMR

**DOI:** 10.1021/acs.analchem.3c05875

**Published:** 2024-06-29

**Authors:** Mark I. Grimes, Matthew Cheeks, Jennifer Smith, Fabio Zurlo, Mick D. Mantle

**Affiliations:** † Department of Chemical Engineering and Biotechnology, University of Cambridge, Philippa Fawcett Drive, Cambridge CB3 0AS, U.K.; ‡ Cell Culture & Fermentation Sciences, Biopharmaceutical Development, Biopharmaceuticals R&D, AstraZeneca, Francis Crick Avenue, Cambridge CB2 0AA, U.K.

## Abstract

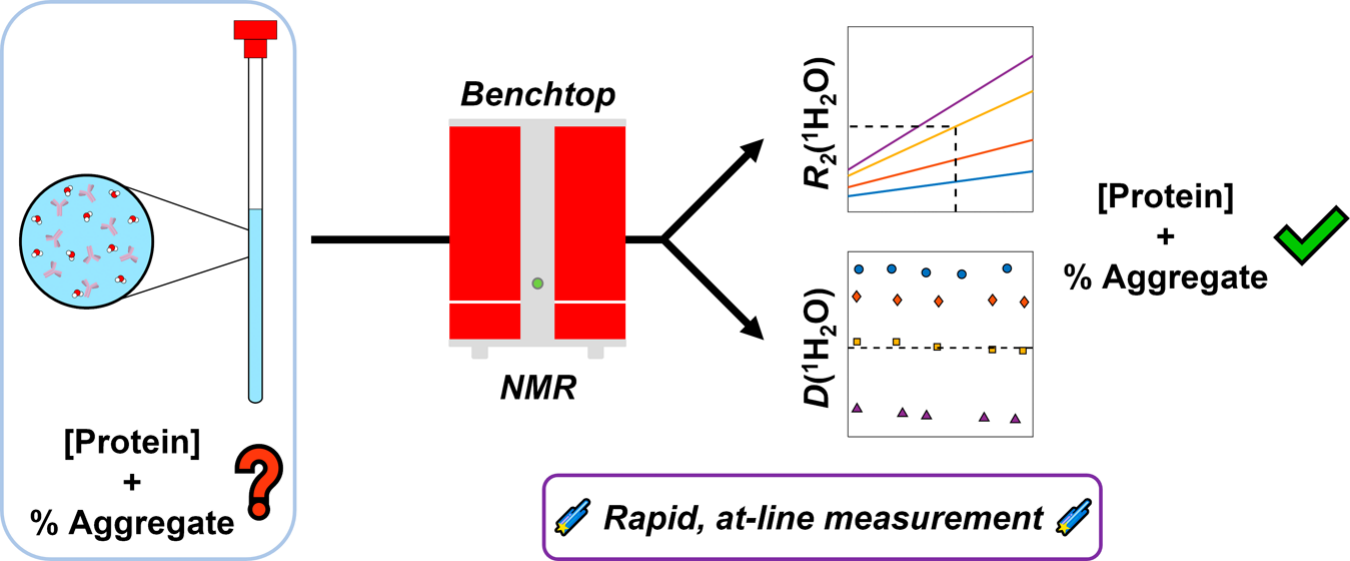

Protein-based biopharmaceutical drugs, such as monoclonal antibodies, account for the majority of the best-selling drugs globally in recent years. For bioprocesses, key performance indicators are the concentration and aggregate level for the product being produced. In water NMR (*w*NMR), the use of the water transverse relaxation rate [*R*_2_(^1^H_2_O)] has been previously used to determine protein concentration and aggregate level; however, it cannot be used to separate between them without using an additional technique. This work shows that it is possible to “decouple” these two key characteristics by recording the water diffusion coefficient [*D*(^1^H_2_O)] in conjunction with *R*_2_(^1^H_2_O), even in the event of overlap in either *D*(^1^H_2_O) or *R*_2_(^1^H_2_O). This method is demonstrated on three different systems, following appropriate *D*(^1^H_2_O) or *R*_2_(^1^H_2_O) calibration data acquisition for a protein of interest. Our method highlights the potential use of benchtop NMR as an at-line process analytical technique.

## Introduction

Biopharmaceuticals can be defined as therapeutic drugs that are produced in biological sources using biotechnological processes.^1^ Examples include hormones and proteins, such as monoclonal antibodies (mAbs). Of these, mAbs accounted for 50% of the top ten best-selling drugs in 2020, with sales of $59 billion,^2^ and more than 800 are currently being studied in clinical trials.^3^ They are a well-established drug class with a higher success rate than small-molecule drugs in receiving regulatory approval since their first approval in 1986.^4,5^ The next generation of antibody-based therapeutics being developed includes bispecific antibodies (BisAbs), which are capable of targeting two different antigens at once, compared to the single antigen that mAbs are able to target. For example, by being able to bind to a target of choice, while simultaneously recruiting cytotoxic immune cells, BisAbs may potentially result in fewer side effects in patients as a result of their enhanced functionality.^6^

Modern biopharmaceuticals are typically produced in bioreactors, which range in size from 1 to 20,000 L, depending on the stage of development.^7,8^ Within bioreactors, the level of product aggregation is monitored and strictly controlled as a part of product quality;^9^ this is because aggregates can cause an increase in the risk of an immune system response in patients.^10^ The mechanisms behind protein aggregation consist of a multitude of different pathways, which can differ between proteins; as such, certain proteins may have high susceptibility to aggregation, while others are more resistant. These different pathways can result in numerous different types of aggregates with the potential to form soluble and/or insoluble aggregates. Insoluble aggregates may also have differing morphologies, which can be influenced by the protein and its environment.^11^ Once aggregated, a protein cannot typically be returned to its native state, meaning that it must be removed from the final drug product in a downstream purification process. This will also reduce the amount of product present (the titer) in the bioreactor, reducing the efficiency of the overall bioprocess. Product aggregation is a common problem in the production of BisAbs, as issues with the mispairing of engineered disulfide bonds can arise due to enzyme-induced reduction.^12,13^ The titer and aggregate level both form key process performance indicators of a large cell culture process, which must meet predefined criteria to be measured as successful.^14^

To determine bioprocess titer, methods such as high-performance liquid chromatography (HPLC) are used.^15^ However, this is normally done as an offline measurement, and HPLC systems have a high capital cost, with specialized training required to operate them. The traditional method for aggregate monitoring is size-exclusion chromatography (SEC). Techniques such as dynamic light scattering (DLS)^16,17^ or mid-infrared spectroscopy^18^ have also been used. However, these methods either require offline analysis, as is the case for SEC, or may potentially be affected by the absorption of water, as it is the major component (>90% w/w) of cell culture media.^19^ Furthermore, no single technique is capable of studying the full-size range or types of possible protein aggregates, with individual methods offering specific advantages as well as limitations.^11,20^ For optical methods, the turbid nature of late-stage cell culture may also provide issues with quantitative analysis due to multiple light scattering as a result of cells and particles in the media.^21^

Water proton nuclear magnetic resonance (*w*NMR) makes use of the transverse relaxation rate of the water signal [*R*_2_(^1^H_2_O)] to gain an understanding about solutes. With water making up the majority of cell culture media/biopharmaceutical samples, the use of the water signal eliminates the need for water signal suppression, which can often cause the loss of sensitivity for the desired analyte as a result of signal loss.^22^ As a noninvasive and nondestructive technique, a wide variety of samples can be analyzed. Furthermore, the turbidity of the sample does not influence the sample analysis. To date, it has been utilized in numerous biopharmaceutical-related studies, such as to investigate the gene filling of adeno-associated viral capsids,^23^ monitor vaccine adjuvant filling level,^24^ and sedimentation kinetics,^25^ in addition to the inspection of biologic product vials.^26−28^ With respect to proteins, *w*NMR has been used to monitor the oxidation of a mAb in real time,^29^ as well as to obtain information about solution protein concentration under both static^30^ and flow conditions,^31^ with sensitivity shown to concentrations as low as 0.4 mg mL^–1^.^32^ Taraban et al. have also demonstrated that *R*_2_(^1^H_2_O) is sensitive to protein aggregates with a size smaller than 0.45 μm and showed that it was capable of outperforming SEC, DLS, and microflow imaging.^33^ It has also been reported that *R*_2_(^1^H_2_O) can be used to characterize aggregates caused by different physical stresses.^34^

A major factor in determining the sensitivity of *R*_2_(^1^H_2_O) in protein solutions is fast chemical exchange between water and protein protons.^35^ The addition of protein to aqueous solutions also results in an increase in the rotational correlation time (the time taken for a water molecule to rotate one radian), which consequently causes an increase in the relaxation rate of water.^36^ The formation of aggregates causes an increase in *R*_2_(^1^H_2_O) because of less efficient averaging of dipole–dipole interactions between protein protons by rotational motions, which impacts water protons as a result of chemical exchange.^37^ Overall, the field strength of the spectrometer used impacts the sensitivity of *R*_2_(^1^H_2_O), with a reduction in sensitivity at lower field strengths.^38^

While having higher sensitivity is an advantage, high-field (>200 MHz for ^1^H) NMR spectrometers are expensive, have a large physical footprint, and require cryogens to maintain the superconducting magnet. As such, they are unfeasible to use at or on a production line. The improvement of permanent magnet design and technology has allowed for the creation of low-field, high-resolution NMR spectrometers (also known as benchtop spectrometers). These use a Halbach array, giving a homogeneous magnetic field that does not stray outside the magnet.^39−41^ They allow for a much more compact design than high-field spectrometers, meaning they can be used in more accessible areas rather than specialized facilities, as well as being more portable. The previously described *R*_2_(^1^H_2_O) studies have largely used low-field NMR, with no penalty from the reduced sensitivity.

A weakness of using *R*_2_(^1^H_2_O) in the study of protein solutions is that it cannot differentiate between the concentration and aggregate content. *R*_2_(^1^H_2_O) has been shown to be influenced by both, and, to date, it has not been successfully used as a standalone analytical technique for these systems.^42^ Instead, additional tests such as UV–vis spectroscopy must be used to discern between the concentration and aggregate content.

This work aims to extend *w*NMR by combining low-field NMR relaxometry and diffusometry to create a method by which aggregate content and protein concentration can be unambiguously differentiated, which we have named Diffusion-Relaxation water NMR (DR*w*NMR). By investigating protein systems ranging in molecular weight up to approximately 150 kDa, we demonstrate the applicability of DR*w*NMR to those widely used in modern biopharmaceuticals. By solely investigating the behavior of the water signal, we eliminate the need for complex sample preparation or the addition of water signal suppression techniques to NMR experiments. The use of a low-field (benchtop) NMR spectrometer enables this method to be utilized in settings outside of a traditional analytical laboratory, such as at the production line, while short experiment times allow for rapid analysis of samples. This method will extend the capability of NMR to study biopharmaceutical samples in a noninvasive manner, broadening the list of potential applications, as well as acting as a foundation from which the complexity of samples analyzed can be increased, with the ultimate goal being the study of cell culture within a bioreactor.

## Experimental Section

Bovine serum albumin (BSA, heat shock fraction, pH 7, ≥98%) was obtained from Sigma-Aldrich (UK). Nonsterile deionized water and phosphate-buffered saline (PBS) were obtained from the departmental media supply at the Department of Chemical Engineering and Biotechnology, University of Cambridge, UK. mAb, a monoclonal antibody, and BisAb, a bispecific antibody, were produced and purified at AstraZeneca (Cambridge, UK). Both were provided in the form of a concentrate, with nominal concentrations of 51.6 and 10 mg mL^–1^, respectively.

Protein solutions were made at the desired concentration by either dissolving BSA in PBS or by diluting the relevant antibody concentrate to the desired concentration using H_2_O. Concentrations between 2 and 10 mg mL^–1^ were investigated, with the upper value reflecting the maximum antibody titers found in fed-batch bioreactors.^14^ The created solutions were then split into two portions; one was placed into a preheated water bath at 65 °C (stressed) and the other stored at 4 °C (unstressed). After a period of time (3 h for BSA solutions; 18 h for mAb solutions), the solutions were taken and allowed to return to room temperature. Solutions with differing stressed fraction levels were made up by taking an aliquot of stressed solution and “diluting” using the unstressed solution (see Figure S1 for a schematic). Due to material constraints, BisAb solutions with a volume of *ca*. 0.5 mL were made and transferred to 5 mm NMR tubes (Wilmad, US; obtained from Sigma-Aldrich, UK). After NMR analysis, the solutions were heated at 65 °C for 18 h in the NMR tubes in a water bath; after removal from the water bath, the same solutions were analyzed again.

The concentration of all protein solutions was verified using a ND-1000 spectrophotometer (NanoDrop Technologies, US) by recording the ultraviolet (UV) absorption at 280 nm (values can be found in Table S1). All measurements were recorded in quintuplicate, with the arithmetic mean value taken to give the sample concentration and the standard error of this concentration used to determine sample error. The aggregate percentage was determined using SEC. SEC analysis was performed with an ÄKTA Pure FPLC system (GE Healthcare, Sweden). Samples were loaded onto a HiLoad 16/60 Superdex 200 prep grade column and eluted using PBS at a flow rate of 1 mL min^–1^; each run was carried out at ambient temperature within a temperature-controlled laboratory (typically 21.5 ± 1.0 °C). Samples were stored at 4 °C and warmed to ambient temperature immediately prior to analysis. The elution profile was monitored by UV absorption at 280 nm. SEC analysis was not performed on all BisAb samples.

For the BSA samples, the aggregate percentage was determined by taking the area percentage for the relevant peak(s) from the acquired chromatogram. To calculate the aggregate percentage for each mAb sample, the built-in Curve Fitting Tool in MATLAB 2022a (MathWorks, US) was used to generate a Gaussian fit for the protein peaks with multiple components used if required. After this, the aggregate content was determined by integrating solely the Gaussian curve that matched the monomer peak, as well as the whole area of the SEC chromatograms and calculating the ratio using a custom script in MATLAB 2022a (MathWorks, US). Known column artifacts or nonprotein peaks were excluded from the integral determination, with protein oligomers (where present) counted as aggregates for the purposes of this work. Aggregate percentages and SEC chromatograms for BSA and mAb samples, as well as the generated fits and residual plots for mAb samples, can be found in the Supporting Information.

DLS analysis was carried out using a NanoBrook Omni (Brookhaven, US), using 10 mm glass cuvettes and a scattering angle of 90°. The instrument was controlled by using BIC Particle Solutions (Brookhaven, US). Samples were passed through a 0.22 μm syringe filter and then analyzed at 25 °C (after equilibrating for 60 s) using a viscosity and refractive index of 0.890 cP and 1.333, respectively. Measurements were carried out in quintuplicate, and the arithmetic mean was used to determine the average effective diameter (values can be found in Table S4).

NMR data was obtained using a 1 T Spinsolve spectrometer (Magritek, Germany), operating at a ^1^H frequency of 43 MHz, which was controlled through Spinsolve Expert v1.41.20 (Magritek, Germany). The spectrometer is equipped with a gradient coil capable of providing a maximum theoretical gradient strength of 107 mT m^–1^. Prior to analysis, all samples were placed into 5 mm NMR tubes (Wilmad, US; obtained from Sigma-Aldrich, UK) and allowed to equilibrate to the magnet temperature (28.50 °C) for at least 5 min. Once this time had elapsed, on-resonance condition check, ^1^H spectral acquisition, and batch folder setup were carried out prior to commencing relaxation and diffusion experiments.

To determine the water transverse relaxation time [*T*_2_(^1^H_2_O)] and diffusion coefficient [*D*(^1^H_2_O)], the “T2Bulk” and “PGSE” pulse sequences were used, respectively. These are provided by the manufacturer and were used without modification. The repetition time was kept at 10 s, with 4 scans accumulated.

The “T2Bulk” pulse sequence is a “one-shot” Carr–Purcell–Meiboom–Gill (CPMG) sequence,^43^ where only the decay of the signal is recorded (i.e., no spectral data is taken). To obtain the transverse relaxation rate, the transverse relaxation time (*T*_2_) for the water signal is first extracted by fitting the data to eq 1
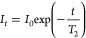
1where *I*_*t*_ is the signal intensity at time *t*, and *I*_0_ is the signal intensity at *t* = 0. A single exponential fit was used to give *T*_2_(^1^H_2_O), the reciprocal of which gives *R*_2_(^1^H_2_O). In these experiments, the following parameters were used: 20 μs radiofrequency (RF) pulse, with a 90 and 180° amplitude of −6 and 0 dB, respectively; 1 μs dwell time; 32 points; and 8192 echoes, with an echo time of 2000 μs. The total experiment time for one measurement was approximately 2 min. Example data is shown in Figure S15.

The “PGSE” pulse sequence is a pulsed gradient spin echo experiment.^44^ To extract the diffusion coefficient (*D*), the data is fitted to eq 2
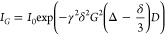
2where *I*_*G*_ is the signal intensity at gradient strength *G*, *I*_0_ is the signal intensity at *G* = 0, γ is the gyromagnetic ratio (2.675 × 10^8^ rad s^–1^ T^–1^ for ^1^H), and δ and Δ are the duration and separation, respectively, of the gradient pulses applied with gradient strength *G*. A single exponential fit was used to give *D*(^1^H_2_O). Values for fitting were generated by integrating over the entire spectrum; in the event that other peaks were present that interfered with the integration, only the water peak was integrated. For all experiments, the following parameters were used: 8.7 μs RF pulse, with a 90° amplitude of 0 dB; δ of 5 ms; Δ of 100 ms; 200 μs dwell time; 16384 points; and 8 gradient steps from 17.8 mT m^–1^ up to a maximum *G* of 107 mT m^–1^. The total experiment time for one measurement was approximately 6 min. Example data are shown in Figures S16–S18.

All NMR data was processed, analyzed, and visualized in MATLAB R2020b or R2022a (MathWorks, US), using custom scripts. Linear fits were also applied in MATLAB. All NMR measurements were recorded in triplicate, with the arithmetic mean value taken to give the sample value; the standard error of this value was used to determine sample error. Values for *R*_2_(^1^H_2_O) and *D*(^1^H_2_O) for all proteins studied, as well as their associated error, can be found in the Supporting Information.

## Results and Discussion

**Figure 1:**
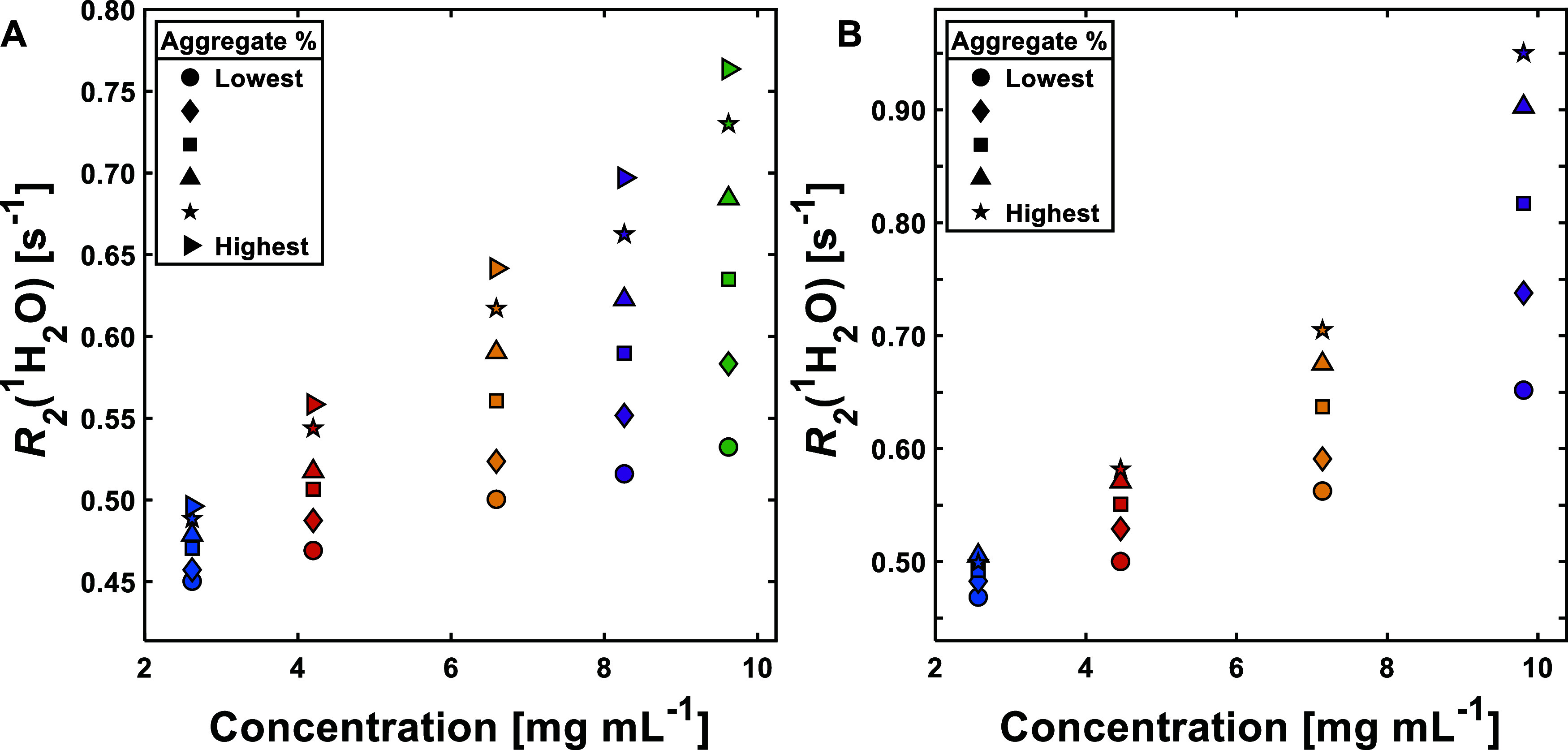
Plots showing the effect of the concentration on *R*_2_(^1^H_2_O) for BSA (A) and mAb (B) solutions. The colors represent individual concentrations studied, while different plot markers represent changing aggregate contents from lowest to highest within each concentration (for exact aggregate content values, see Tables S2 and S3). Sample errors were determined by taking the standard error of the arithmetic mean of three sample measurements; values can be found in Tables S2 and S3. Error bars are excluded for clarity where the errors are smaller than the symbols used.

Initial studies focused on investigating the effect of changing both protein concentration and aggregate content on *R*_2_(^1^H_2_O), with a view to eventually increasing in complexity toward cell culture. *R*_2_(^1^H_2_O) is known to have a linear dependence with each,^42^ but it is unclear what their combined effect would be, particularly as aggregate content is varied. When both aggregate content and BSA concentration are increased, it can be observed that these two characteristics combine together to give increased sensitivity when plotted against the concentration (Figure 1A). Out of the two factors, increasing the aggregate content gives the largest contribution to the overall effect, causing an increasing dispersion of *R*_2_(^1^H_2_O) values with increasing concentration. The same trend is observed with mAb solutions (Figure 1B).

**Figure 2:**
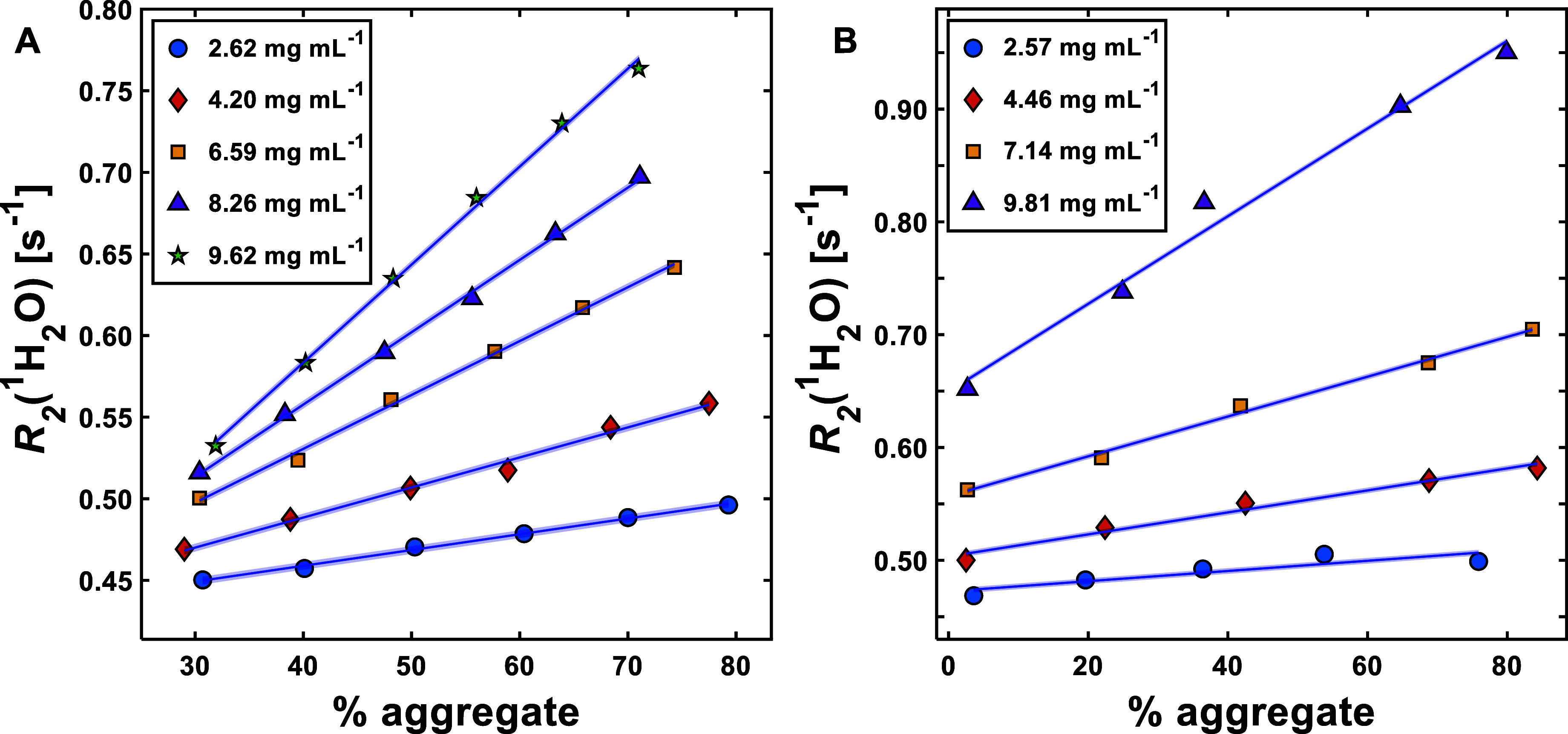
Plots showing the effect of aggregate percentage on *R*_2_(^1^H_2_O) for BSA (A) and mAb (B) solutions over a range of different concentrations. The solid lines represent linear regression fits to individual sample concentrations (as determined in MATLAB 2022a [MathWorks, US]), and the blue-shaded areas represent 95% confidence intervals calculated from average of the standard error of all data points for each protein. The 95% confidence intervals are multiplied by a factor of 5 for visibility. Sample errors were determined by taking the standard error of the arithmetic mean of three sample measurements; values can be found in the Tables S2 and S3. Error bars are excluded for clarity where the errors are smaller than the symbols used.

**Figure 3:**
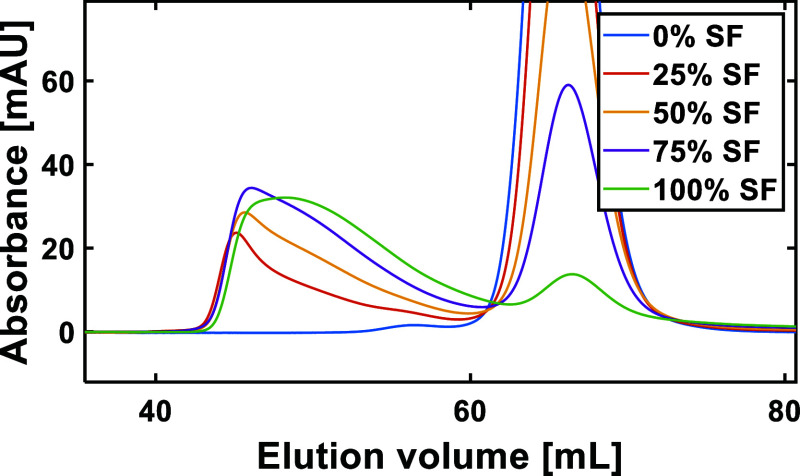
Section of a SEC chromatogram recorded for 9.81 mg mL^–1^ mAb solutions, with varying stressed fractions (SF) between 0 and 100%.

Figure 2 shows individual concentrations plotted against known aggregate content for both BSA and mAb. Here, linear trends are observed for both proteins, with clear separations between different concentrations. With the exception of the 2.57 mg mL^–1^ mAb solution, the *R*^2^ values for the linear fits shown in Figure 2 were all greater than 0.98. At the highest aggregate percentage values for mAb, it can be observed that *R*_2_(^1^H_2_O) slightly deviates from the expected trend. This is particularly apparent with the 2.57 mg mL^–1^ solution, where the value of *R*_2_(^1^H_2_O) with 75.9% aggregate content is actually less than that with 53.8% aggregate content. This explains the poor *R*^2^ value for this data set, compared to others. Despite this, at higher concentrations, *R*_2_(^1^H_2_O) is always at its highest value at the highest aggregate percentage. Upon further inspection of the SEC chromatograms for the mAb solutions, it can be seen that the aggregate peak shifts to a higher elution volume with increased stressed fraction content (Figure 3). This suggests that aggregate size for mAb is influenced by changing aggregate content.

To verify this, DLS analysis was carried out on a 10.07 mg mL^–1^ mAb solution, with three different stressed fractions (0, 50, and 100%) studied. The average effective diameter of the 0% stressed fraction sample was found to be 8.1 ± 0.1 nm, which matches with the typical hydrodynamic diameter of a monoclonal antibody.^45^ For the 100% stressed fraction sample, the average effective diameter was found to be 26.9 ± 0.1 nm, whereas for the 50% stressed fraction sample, it was 31.6 ± 0.8 nm (see Figure S19 for illustrative size distributions). This confirms that there is a change in aggregate size with increasing aggregate content. Given that *R*_2_(^1^H_2_O) has been shown to be sensitive to aggregates smaller than 0.45 μm,^33^ it is also understandable that the size of the aggregate will also impact *R*_2_(^1^H_2_O), although to a smaller degree than overall protein concentration and aggregate content. At low concentrations, the aggregate size may influence *R*_2_(^1^H_2_O) to a similar level as the protein concentration, which explains the trend in *R*_2_(^1^H_2_O) seen with the 2.57 mg mL^–1^ mAb solution in Figure 2B.

The combined effect of increased aggregate percentage and increasing protein concentration is unsurprising, given the fast chemical exchange between the water solvent and the protein species in solution. Further to this, *R*_2_(^1^H_2_O) is also impacted by the surface water translational correlation time, which is associated with surface diffusion. Kimmich et al. found that the reduced diffusion of water at the protein surface helped to explain the relaxation behavior of protein solutions with concentration.^46^ The surface diffusion of water is shown to be reduced in aggregated protein,^47^ which results in a slowing of the associated correlation time of water molecules and a subsequent increase in *R*_2_(^1^H_2_O). This explains why increasing aggregate content has a larger contribution than concentration in the changing sensitivity of *R*_2_(^1^H_2_O).

Further to this, an investigation into whether the use of the longitudinal relaxation rate [*R*_1_(^1^H_2_O)] could be used to differentiate between protein concentration and aggregate content was carried out, in light of Taraban et al. suggesting that this may be possible when used in combination with *R*_2_(^1^H_2_O).^31^ An initial experiment using a BSA solution with a nominal concentration of 12.5 mg mL^–1^ confirmed that *R*_1_(^1^H_2_O) is indeed insensitive to aggregate content (Figure S20), as has been shown previously.^48^ However, while no significant variation with aggregate content was recorded, there was little to no difference between increasing concentrations at 43 MHz; in fact, in some instances, *R*_1_(^1^H_2_O) was seen to be lower for higher concentrations of BSA (Figure S21). This goes against the anticipated trend, where *R*_1_(^1^H_2_O) is expected to have a dependence with protein concentration.^49^ As such, it follows that the use of *R*_1_(^1^H_2_O) does not offer the resolution necessary to differentiate between concentration and aggregate content at the magnetic field strength used here. Despite this, *R*_1_(^1^H_2_O) may become more useful at lower field strengths. Bryant et al. have previously shown in a fast-field cycling (FFC) study that below 1 MHz, there is significant divergence in the behavior of *R*_1_(^1^H_2_O) between solutions containing BSA with, or without, cross-linking.^50^ This is explained to be due to the coupling of the water spin relaxation through intermolecular dipole–dipole interactions to that of the solid protein matrix (formed by the cross-linking), which shows magnetic field dependence.

**Figure 4:**
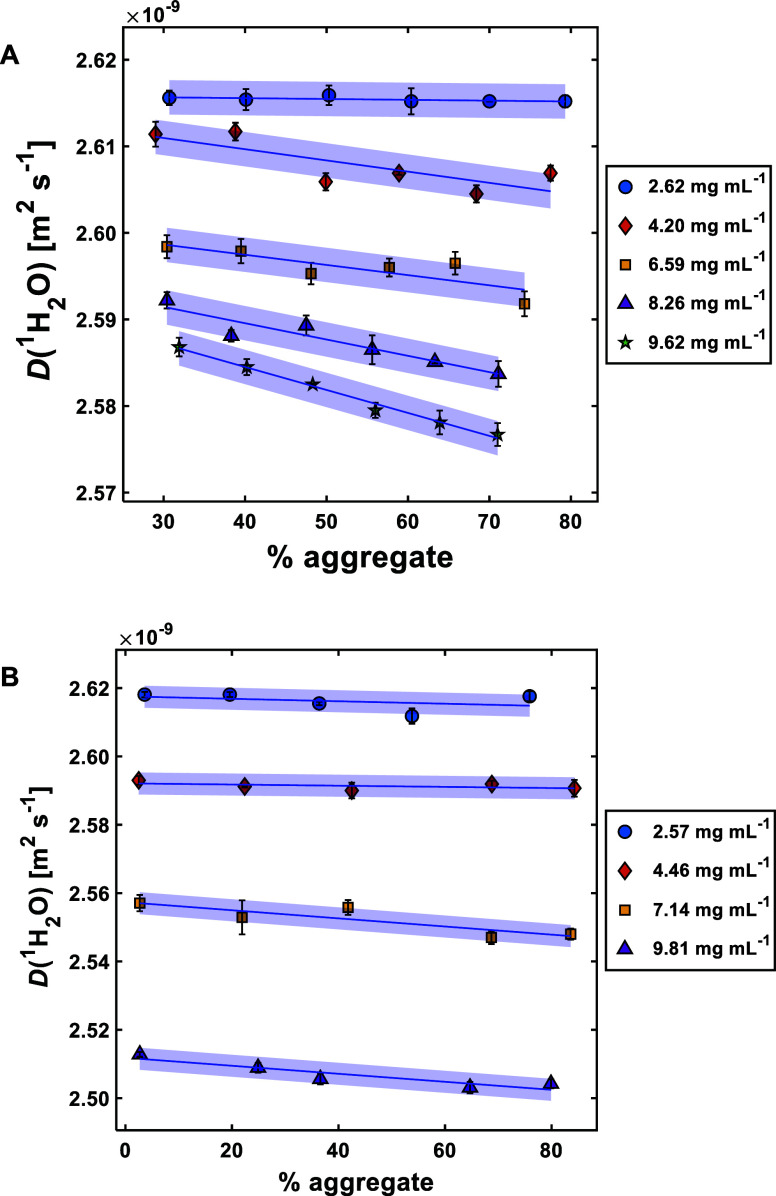
Plots showing the effect of aggregate percentage on *D*(^1^H_2_O) for BSA (A) and mAb (B) solutions over a range of different concentrations. The solid lines represent linear regression fits to individual sample concentrations (as determined in MATLAB 2022a [MathWorks, US]), and the blue-shaded areas represent 95% confidence intervals calculated from average of the standard error of all data points for each protein. Individual data point errors were determined by taking the standard error of the arithmetic mean of three sample measurements; values can be found in Tables S2 and S3.

When looking at data acquired for the water diffusion coefficient [*D*(^1^H_2_O)] in our experiments, it became apparent that this could provide the key to “decoupling” concentration and aggregate content. With increasing protein concentration, *D*(^1^H_2_O) decreases (Figure 4). This is due to the “obstruction effect” identified by Kimmich et al., where solutes (in this case, the protein) impede the diffusion pathways of water molecules.^51^ The hydration of the protein may also be influencing *D*(^1^H_2_O), as a part of the cell-diffusion model described by Jönsson et al.^52^ Here, water–protein interactions and obstruction effects are both taken into account.^53^ Similar reductions in diffusion coefficient with increasing protein concentration have been observed in a whey protein study,^53^ as well as when trifluoroethanol was studied as a probe molecule in BSA solutions.^54^

At higher concentrations for both BSA and mAb, the beginning of a linear dependence can be observed, where the value of *D*(^1^H_2_O) begins to decrease with increasing aggregate content. This is to be expected, as aggregates in the solution will impact the ability of water to diffuse freely more than monomers. A similar reduction in *D*(^1^H_2_O), albeit at a larger magnitude, was observed after calcium chloride had been used to induce the formation of aggregates in soybean protein dispersions by Hong and Lee.^55^ For BSA, some overlap in *D*(^1^H_2_O) values can be observed, particularly at higher concentrations (see Figure 4A, 8.26 and 9.62 mg mL^–1^). However, mAb solutions show increased sensitivity in *D*(^1^H_2_O) compared to BSA, with no overlap observed, regardless of concentration. This is due to *D*(^1^H_2_O) being mainly affected by protein concentration rather than the aggregate state.^56^ To the best of the authors’ knowledge, there has been no previous work studying the change in water diffusion coefficient with varying aggregate content.

**Figure 5:**
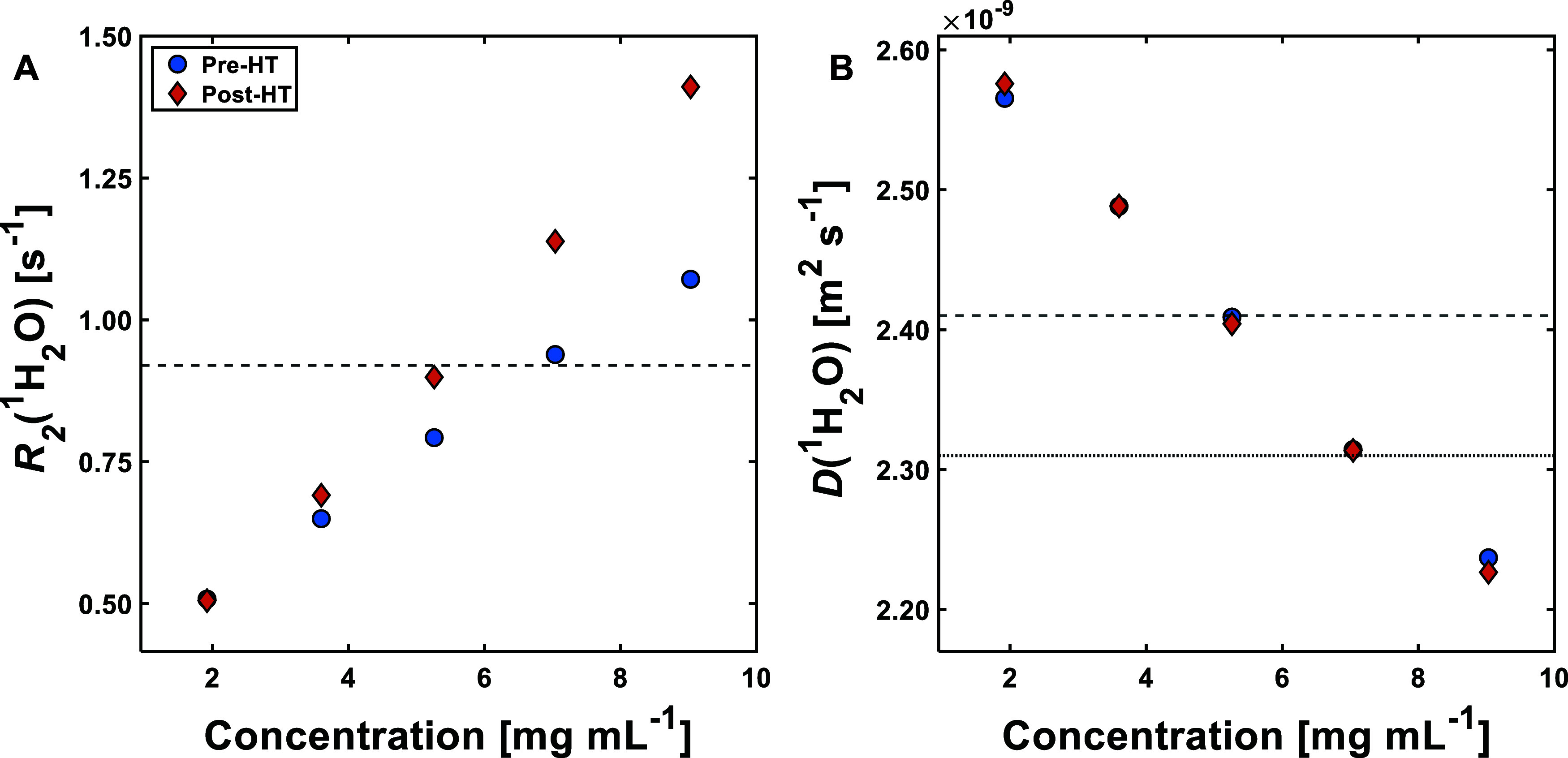
Plots showing the effect of concentration with *R*_2_(^1^H_2_O) (A) and *D*(^1^H_2_O) (B) for BisAb solutions before (Pre-HT, circles) and after heat-stress (Post-HT, diamonds). The dashed line in (A) represents a constant *R*_2_(^1^H_2_O) value of 0.920 s^–1^. The dotted and dashed lines in (B) represent constant *D*(^1^H_2_O) values of 2.310 × 10^–9^ and 2.410 × 10^–9^ m^2^ s^–1^, respectively. Sample errors were determined by taking the standard error of the arithmetic mean of three sample measurements; values can be found in Table S5. Error bars are excluded for clarity, where the errors are smaller than the symbols used.

Due to material constraints, BisAb solutions were only investigated before and after heat-stressing. Figure 5 shows the effect of concentration with *R*_2_(^1^H_2_O) and *D*(^1^H_2_O) for BisAb solutions before and after heat-stress (equivalent plots for BSA and mAb are presented as Figures S26 and S27, respectively). Comparing the two sets of data, it is apparent that while *R*_2_(^1^H_2_O) values have changed with heat-stressing, *D*(^1^H_2_O) has not changed significantly. The change in *R*_2_(^1^H_2_O) suggests the formation of aggregates, which is confirmed by SEC analysis (Figure S22). Comparing Figures 4 and 5, it is apparent that *D*(^1^H_2_O) shows a larger change over a similar concentration range for BisAb than both BSA and mAb. This is likely to be due to the differences in size between BisAb and BSA, resulting in increased interactions between water and the protein. In the case of BisAb and mAb, it could be that there are more polar/interacting surface residues in BisAb due to the additional binding site present and/or differences in structure. Due to the use of proprietary materials, the exact amino acid sequence for the antibodies used is not known, so this theory cannot be definitely confirmed. Upon visual inspection of the data in Figure 5, it can be observed that an *R*_2_(^1^H_2_O) value of 0.920 s^–1^ is shared by both the 5.26 mg mL^–1^ solution after heat-stress and the 7.04 mg mL^–1^ solution before heat-stress (Figure 5A). In traditional *w*NMR, where solely *R*_2_(^1^H_2_O) is used, it would be impossible to distinguish between these samples without the use of an additional analytical technique. In DR*w*NMR, *D*(^1^H_2_O) is also recorded for the same samples. Based on this, the identity of the sample can be unambiguously assigned, given the difference in value (2.410 × 10^–9^ m^2^ s^–1^ compared to 2.310 × 10^–9^ m^2^ s^–1^ for the respective samples in question, see Figure 5B). In the case of shared values of *D*(^1^H_2_O) for different concentrations and/or aggregate content, such as with the BSA solutions studied here, by following a similar logic as described above, the identity of the sample can be unambiguously assigned by using *R*_2_(^1^H_2_O) values.

The key message here is that the data presented in Figures 2 and 4 [covering *R*_2_(^1^H_2_O) and *D*(^1^H_2_O) values, respectively] can be used to unambiguously determine both the concentration and percentage aggregate of an unknown solution of a protein of interest. The methodology has been described briefly here for BisAb, but further examples for both BSA and mAb samples as well as a flowchart explaining the ideal workflow envisioned for this method can be found in the Supporting Information. A second key point is that for mAb samples, there is no overlap in *D* values (Figure 4B), and therefore a combination of *D*(^1^H_2_O) and *R*_2_(^1^H_2_O) always produces a unique, unambiguous assignment for the concentration and aggregate content for the mAb system studied here. The technique is being developed for general biopharmaceutical production, and we are confident there will be negligible overlap for mAb-producing cell lines with antibody titers ranging up to those typically seen in a bioreactor.^14^ By now being able to differentiate between aggregate content and concentration solely using NMR, there is no requirement to use an additional or alternative technique. This enables faster analysis of samples, as it eliminates the need to prepare a sample for a different analytical method; instead, the same sample can be analyzed by the required NMR experiments in quick succession.

## Conclusions

The combined use of *D*(^1^H_2_O) and *R*_2_(^1^H_2_O) NMR measurements at low-field in DR*w*NMR provides a means by which it is now possible to “decouple” the concentration and aggregate content of protein solutions, where *w*NMR alone is unable to. This holds true for BSA, in addition to monoclonal and BisAbs, demonstrating that DR*w*NMR can be applied to multiple, diverse protein systems.

We have investigated the effect that both protein concentration and aggregate content have on *R*_2_(^1^H_2_O) with BSA and mAb; it was observed that they combined to give increased sensitivity, something that has not been previously demonstrated in the literature. We have also shown that *D*(^1^H_2_O) shows lower sensitivity to aggregate content than *R*_2_(^1^H_2_O), which can be exploited to unambiguously differentiate between protein samples by recording both values for three different protein systems. In addition, we observed that *R*_2_(^1^H_2_O) was sensitive enough to observe differences in the mAb aggregate size of approximately 5 nm. The use of a low-field NMR spectrometer in this work demonstrates the potential to bring this technique into the laboratory or to wherever it is required. Due to the short experiment time required to determine the necessary values (ca. 8 min in total), we envision that DR*w*NMR will provide a rapid and noninvasive method by which these characteristics can be determined, with minimal sample preparation required.

A limitation is that like *w*NMR, an external analytical method is required initially to generate relevant calibration data for the system of interest; however, this method is envisioned to be a complementary technique to those already being used, increasing the range available. Another potential limitation is that the sensitivity of *R*_2_(^1^H_2_O) and *D*(^1^H_2_O) is protein-dependent. Comparing the data for unstressed and stressed samples in Figures 5, S26, and S27 (for BisAb, BSA, and mAb, respectively), it is clear that *D*(^1^H_2_O) in particular may have a shallow dependence on protein concentration. As such, this is something that must be considered carefully for the system of interest by any user; however, we are confident that the parameters used in this work are suitable.

Although initially carried out with an aim toward studying cell culture, we believe that this methodology could be applied to the study of higher concentrations (based on work by others^54,57^) or other types of protein-containing samples, such as purified drug product or potentially even biological vials, extending the scope of *w*NMR beyond that explored in previous works in the literature. Moving forward, the sensitivity of *R*_2_(^1^H_2_O) to aggregate sizes smaller than those previously studied in the literature^34^ will be investigated. Further to this, it would also be of interest to see if DR*w*NMR is suitable to investigate the effect of changing aggregate size, given that *R*_2_(^1^H_2_O) shows sensitivity to changing aggregate size.^33^ The use of flow conditions (using flow-compensated diffusion measurements^58,59^), as well as increasing the complexity of the systems studied toward cell culture, will also be the subject of future studies.

## Supporting Information Available

Schematic of protocol used to generate stressed fractions; concentration data (for all proteins); water transverse relaxation rate [*R*_2_(^1^H_2_O)] and water diffusion coefficient [*D*(^1^H_2_O)] values (for all proteins); SEC data and chromatograms (for BSA and mAb; chromatogram only for BisAb); fits and residual plots (for mAb only); example NMR data (for all proteins); DLS analysis data; behavior of the water longitudinal relaxation rate [*R*_1_(^1^H_2_O)] with increased stressed fraction for BSA solutions; method flowchart; and case studies (mAb and BSA) and MATLAB code used to generate truth arrays (PDF).
