# Supplementary material for: Decoupling Protein Concentration and Aggregate Content Using Diffusion and Water NMR
Source: Anal Chem. 2024 Jun 29;96(28):11155–62. doi: 10.1021/acs.analchem.3c05875 (PMC11256015; doi:10.1021/acs.analchem.3c05875)
Supplement: Supplementary file 1 — ac3c05875_si_001.pdf [file ac3c05875_si_001.pdf]

Supporting Information for:

Decoupling Protein Concentration and Aggregate Content Using Diffusion and Water NMR

Mark I. Grimes[a], Matthew Cheeks[b], Jennifer Smith[b], Fabio Zurlo[b], Mick D. Mantle[a,*]

[a] Department of Chemical Engineering and Biotechnology, University of Cambridge, Philippa Fawcett Drive, Cambridge, CB3 0AS, United Kingdom

[b] Cell Culture & Fermentation Sciences, Biopharmaceutical Development, Biopharmaceuticals R&D, AstraZeneca, Francis Crick Avenue, Cambridge, CB2 0AA, United Kingdom

* Corresponding author: Prof. Mick D. Mantle; email address: mdm20@cam.ac.uk

Table of Contents

Schematic of protocol to generate stressed fractions S2
Concentrations of protein solutions used S3
Data – BSA S4
  Data table S4
  Size-exclusion chromatograms S4
Data – mAb S7
  Data table S7
  Size-exclusion chromatograms S7
  Fits and residuals plots S9
Example NMR data S13
Dynamic light scattering analysis S15
The behaviour of $R_1(^1H_2O)$ with increased stressed fraction for BSA solutions S16
Data – BisAb S18
  Data table S18
  Size-exclusion chromatogram S18
Method flowchart S19
Case studies S20
  First case study – BSA S20
  Second case study – mAb S22
MATLAB code to generate truth array S24
Comparison of 0% and 100% SF data for BSA and mAb S23
References S24

Schematic of protocol to generate stressed fractions

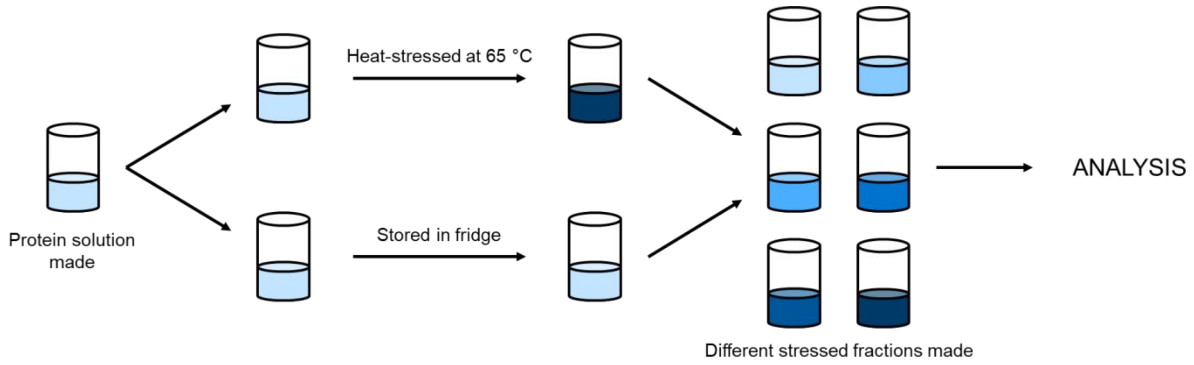

Figure S1 *– Schematic of the protocol followed to generate the different stressed fraction-containing solutions.*

Concentrations of protein solutions used

Table S1 *– Concentrations of the protein solutions studied in this work, as determined by UV-vis spectroscopy.*

Protein	***Concentration / mg mL^{-1}***
BSA	2.62 ± 0.03
	4.20 ± 0.01
	6.59 ± 0.02
	8.26 ± 0.03
	9.62 ± 0.02
mAb	2.57 ± 0.01
	4.46 ± 0.01
	7.14 ± 0.02
	9.81 ± 0.003
BisAb	1.92 ± 0.01
	3.60 ± 0.01
	5.26 ± 0.01
	7.04 ± 0.01
	9.03 ± 0.11

Data – BSA

Data table

Table S2 *– Data for each BSA solution studied in this work. Percentage aggregate content was determined by size-exclusion chromatography analysis, while the water transverse relaxation rate [$R_2(^1H_2O)$] and water diffusion coefficient [$D(^1H_2O)$] were determined by NMR analysis. Sample errors were determined by taking the standard error of the arithmetic mean of three sample measurements.*

Concentration / mg mL^{-1}	Stressed fraction / %	% aggregate	$R_2(^1H_2O)$ / s^{-1}	$D(^1H_2O)$ / × 10^{-9} m^2 s^{-1}
2.62	0	30.7	0.4503 ± 0.0001	2.6156 ± 0.0008
	20	40.1	0.4573 ± 0.0002	2.6154 ± 0.0012
	40	50.3	0.4705 ± 0.0002	2.6159 ± 0.0011
	60	60.4	0.4786 ± 0.0003	2.6152 ± 0.0015
	80	70.0	0.4885 ± 0.0002	2.6152 ± 0.0003
	100	79.3	0.4962 ± 0.0002	2.6152 ± 0.0007
4.20	0	29.0	0.4691 ± 0.0002	2.6114 ± 0.0014
	20	38.8	0.4874 ± 0.0003	2.6117 ± 0.0010
	40	49.9	0.5066 ± 0.0002	2.6059 ± 0.0010
	60	58.9	0.5175 ± 0.0003	2.6069 ± 0.0012
	80	68.4	0.5438 ± 0.0002	2.6045 ± 0.0010
	100	77.5	0.5585 ± 0.0003	2.6069 ± 0.0009
6.59	0	30.4	0.5004 ± 0.0003	2.5984 ± 0.0013
	20	39.5	0.5236 ± 0.0003	2.5979 ± 0.0014
	40	48.1	0.5607 ± 0.0002	2.5953 ± 0.0013
	60	57.7	0.5903 ± 0.0003	2.5960 ± 0.0010
	80	65.8	0.6171 ± 0.0002	2.5965 ± 0.0013
	100	74.3	0.6417 ± 0.0002	2.5918 ± 0.0014
8.26	0	30.4	0.5160 ± 0.0003	2.5922 ± 0.0009
	20	38.3	0.5517 ± 0.0003	2.5881 ± 0.0007
	40	47.5	0.5897 ± 0.0003	2.5893 ± 0.0012
	60	55.6	0.6227 ± 0.0002	2.5865 ± 0.0017
	80	63.3	0.6625 ± 0.0003	2.5851 ± 0.0002
	100	71.1	0.6971 ± 0.0002	2.5837 ± 0.0015
9.62	0	31.9	0.5324 ± 0.0003	2.5868 ± 0.0011
	20	40.2	0.5833 ± 0.0003	2.5845 ± 0.0009
	40	48.3	0.6349 ± 0.0003	2.5825 ± 0.0001
	60	56.0	0.6844 ± 0.0003	2.5795 ± 0.0009
	80	63.9	0.7300 ± 0.0002	2.5781 ± 0.0014
	100	71.0	0.7636 ± 0.0004	2.5767 ± 0.0013

Size-exclusion chromatograms

The peak at an elution volume of approximately 25 mL is a column artefact, and was excluded from any calculations used to determine the percentage monomer content.

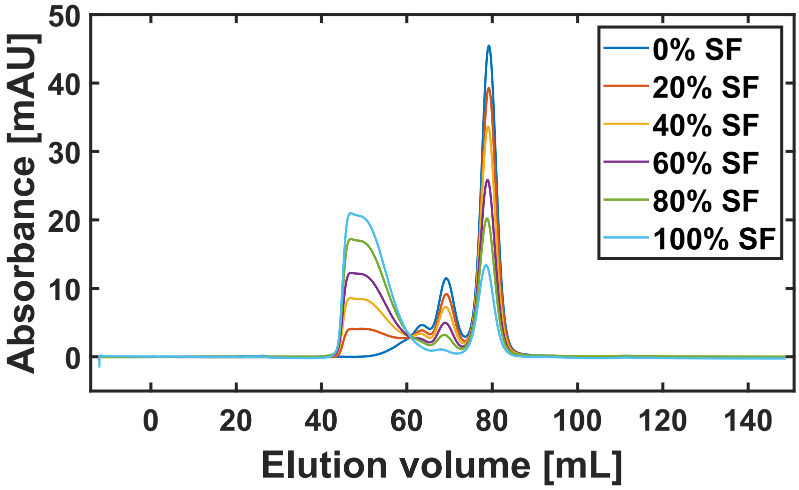

Figure S2 *– Stacked size-exclusion chromatograms recorded for 2.62 mg mL^{-1} BSA solutions at various stressed fractions.*

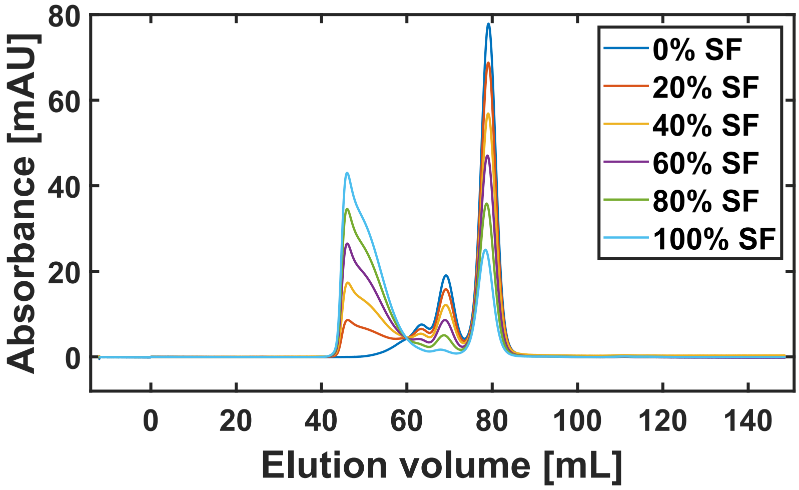

Figure S3 *– Stacked size-exclusion chromatograms recorded for 4.20 mg mL^{-1} BSA solutions at various stressed fractions.*

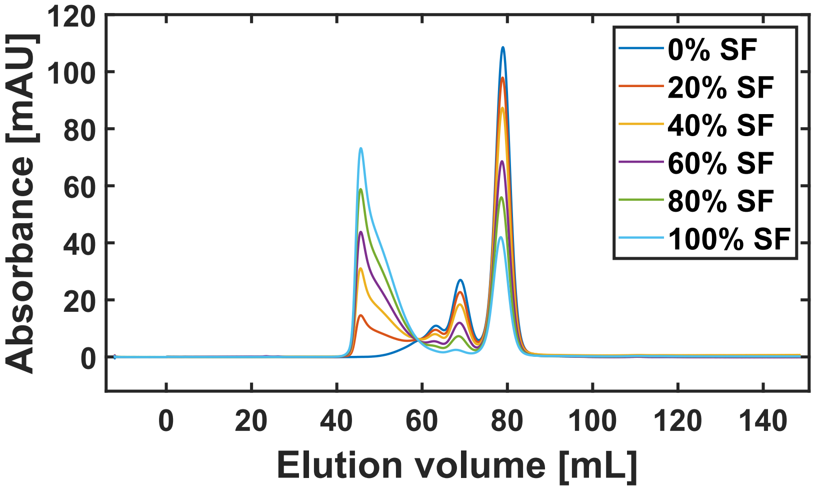

Figure S4 *– Stacked size-exclusion chromatograms recorded for 6.59 mg mL^{-1} BSA solutions at various stressed fractions.*

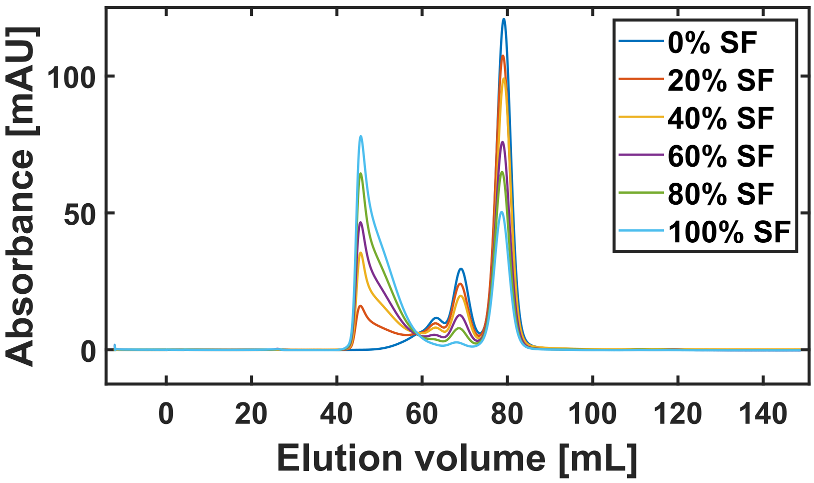

Figure S5 *– Stacked size-exclusion chromatograms recorded for 8.26 mg mL^{-1} BSA solutions at various stressed fractions.*

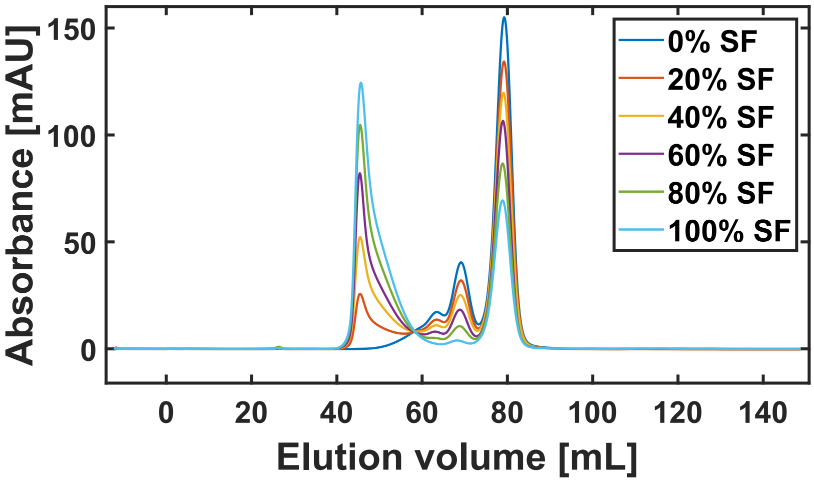

Figure S6 *– Stacked size-exclusion chromatograms recorded for 9.62 mg mL^{-1} BSA solutions at various stressed fractions.*

Data – mAb

Data table

Table S3 *– Data for each mAb solution studied in this work. Percentage aggregate content was determined by size-exclusion chromatography analysis, while the water transverse relaxation rate [$R_2(^1H_2O)$] and water diffusion coefficient [$D(^1H_2O)$] were determined by NMR analysis. Sample errors were determined by taking the standard error of the arithmetic mean of three sample measurements.*

Concentration / mg mL^{-1}	Stressed fraction / %	% aggregate	$R_2(^1H_2O)$ / s^{-1}	$D(^1H_2O)$ / × 10^{-9} m^2 s^{-1}
2.57	0	3.6	0.4686 ± 0.0002	2.6181 ± 0.0008
	25	19.6	0.4826 ± 0.0003	2.6181 ± 0.0007
	50	36.4	0.4923 ± 0.0002	2.6155 ± 0.0005
	75	53.8	0.5053 ± 0.0002	2.6118 ± 0.0022
	100	75.9	0.4990 ± 0.0002	2.6176 ± 0.0015
4.46	0	2.5	0.5001 ± 0.0001	2.5930 ± 0.0013
	25	22.4	0.5290 ± 0.0002	2.5912 ± 0.0003
	50	42.5	0.5507 ± 0.0003	2.5900 ± 0.0023
	75	68.8	0.5708 ± 0.0003	2.5919 ± 0.0010
	100	84.3	0.5818 ± 0.0003	2.5907 ± 0.0025
7.14	0	2.7	0.5626 ± 0.0003	2.5571 ± 0.0024
	25	21.9	0.5909 ± 0.0003	2.5529 ± 0.0050
	50	41.8	0.6370 ± 0.0002	2.5558 ± 0.0022
	75	68.7	0.6751 ± 0.0003	2.5470 ± 0.0019
	100	83.6	0.7049 ± 0.0003	2.5480 ± 0.0016
9.81	0	2.7	0.6518 ± 0.0003	2.5128 ± 0.0007
	25	24.9	0.7378 ± 0.0003	2.5089 ± 0.0014
	50	36.6	0.8171 ± 0.0003	2.5056 ± 0.0016
	75	64.7	0.9027 ± 0.0002	2.5031 ± 0.0017
	100	79.9	0.9502 ± 0.0004	2.5042 ± 0.0013

Size-exclusion chromatograms

The peak at an elution volume of approximately 25 mL is a column artefact; the peak at approximately 120 mL is histidine, a non-protein component of the mAb concentrate used. These were both excluded from any integral calculations used to determine the percentage monomer content.

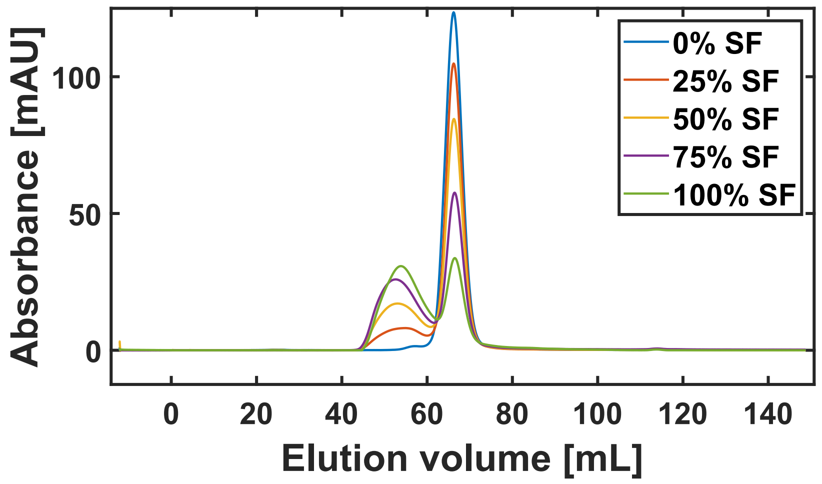

Figure S7 *– Stacked size-exclusion chromatograms recorded for 2.57 mg mL^{-1} mAb solutions at various stressed fractions.*

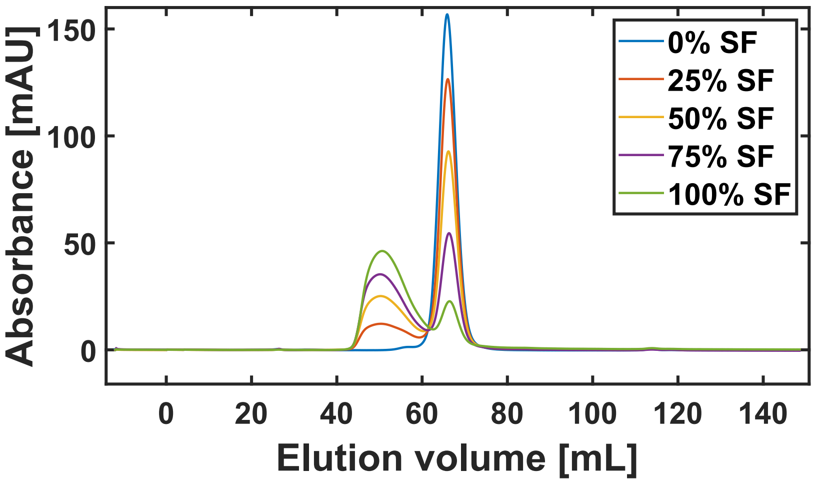

Figure S8 *– Stacked size-exclusion chromatograms recorded for 4.46 mg mL^{-1} mAb solutions at various stressed fractions.*

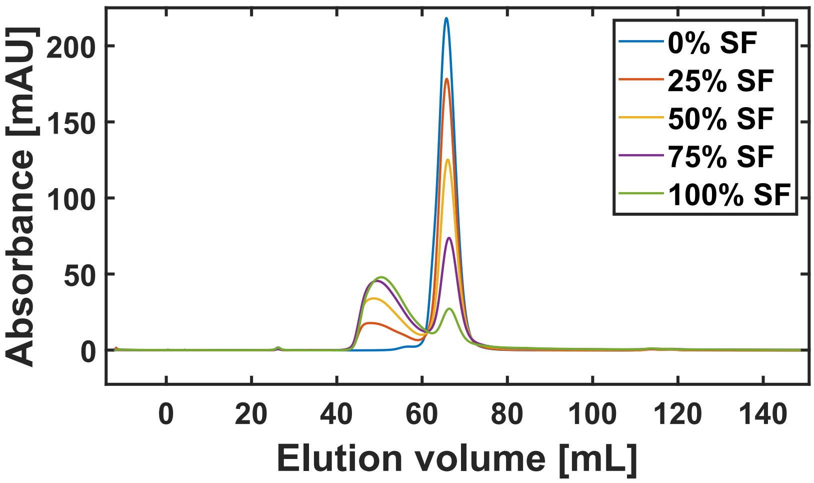

Figure S9 *– Stacked size-exclusion chromatograms recorded for 7.14 mg mL^{-1} mAb solutions at various stressed fractions.*

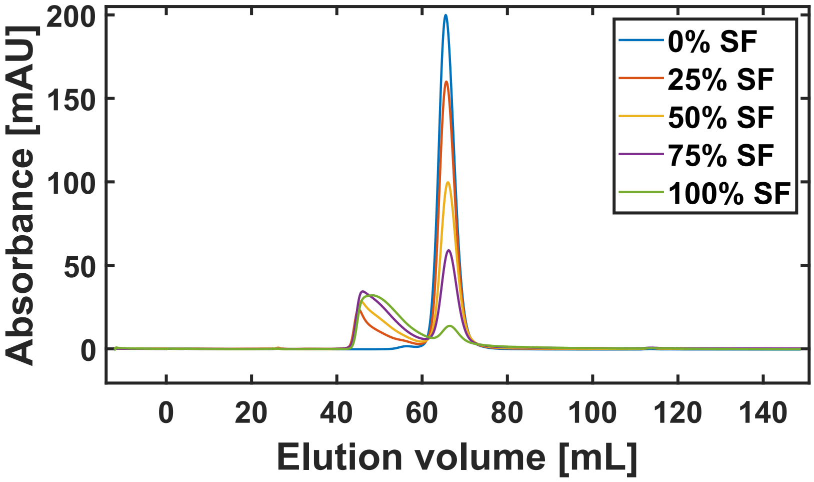

Figure S10 *– Stacked size-exclusion chromatograms recorded for 9.81 mg mL^{-1} mAb solutions at various stressed fractions.*

Fits and residuals plots

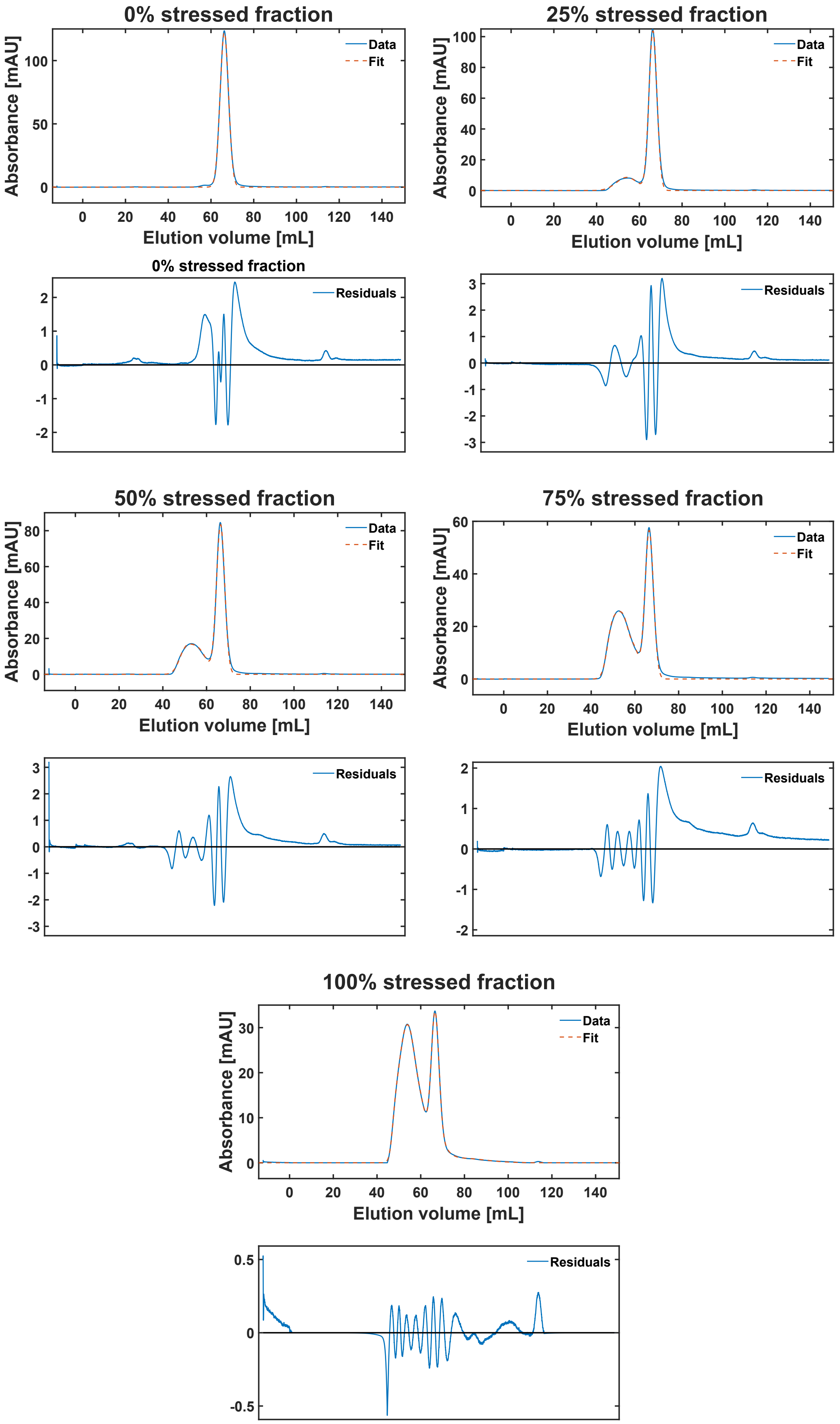

Figure S11 *– Fits and residuals plots for each stressed fraction analysed for a 2.57 mg mL^{-1} mAb solution.*

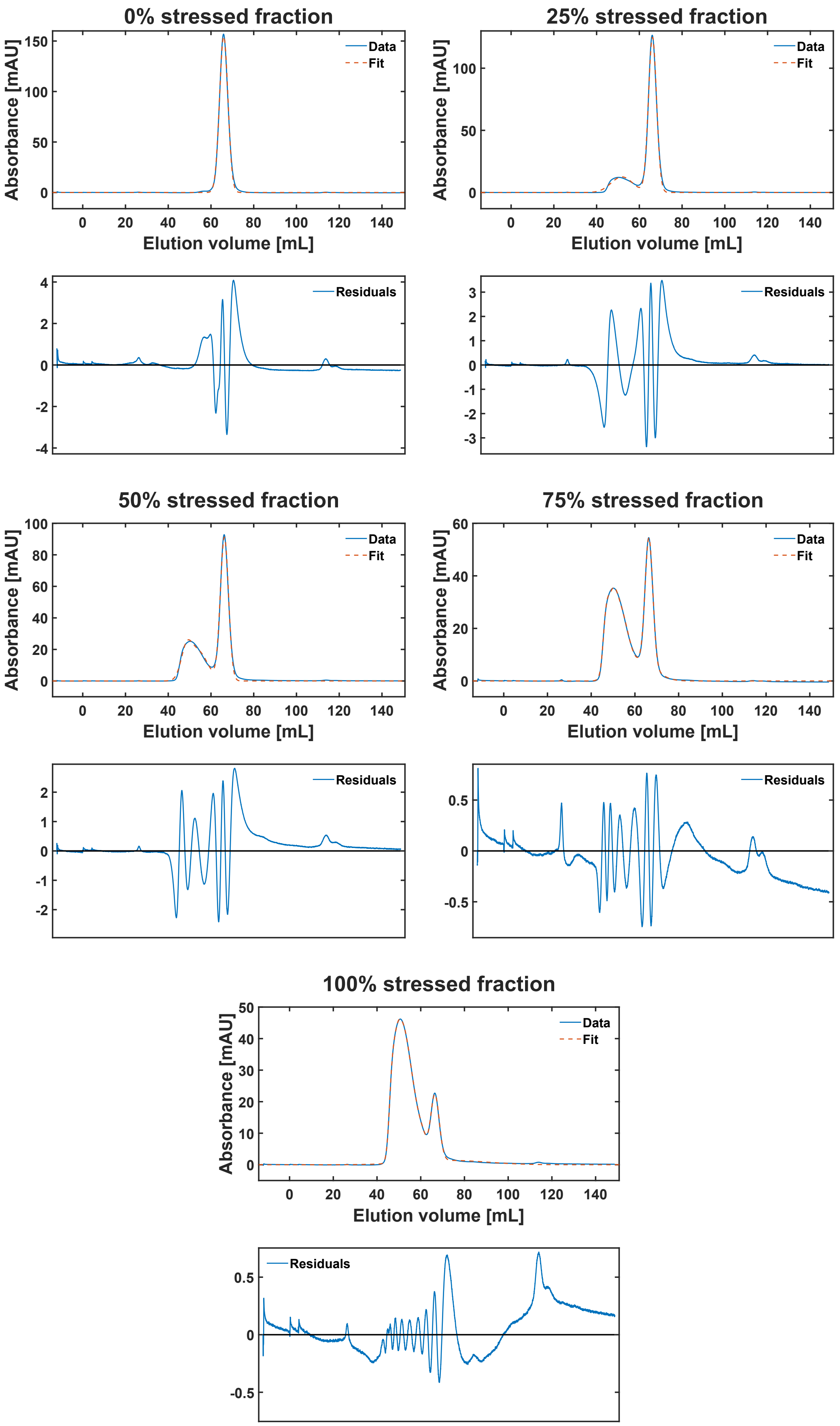

Figure S12 *– Fits and residuals plots for each stressed fraction analysed for a 4.46 mg mL^{-1} mAb solution.*

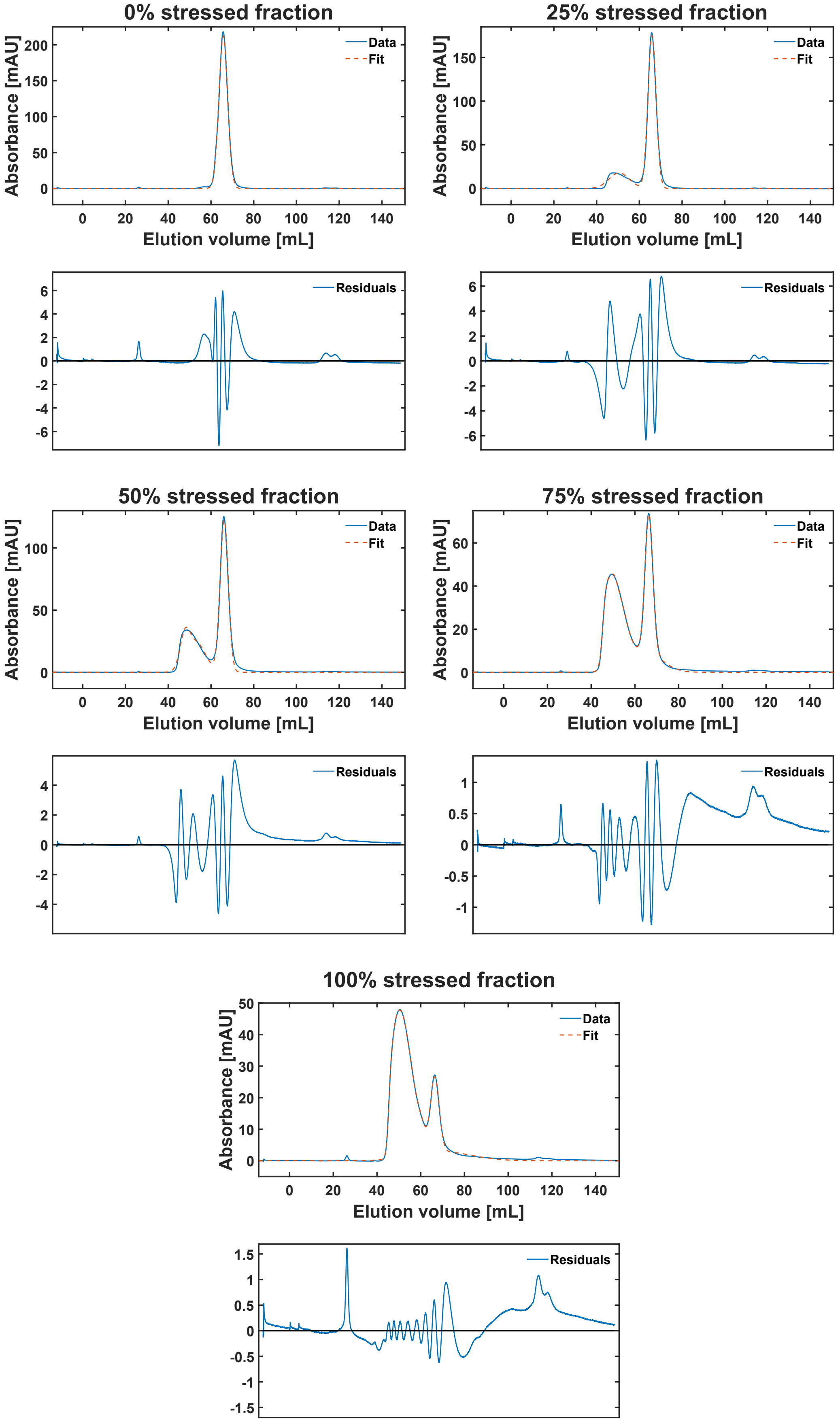

Figure S13 *– Fits and residuals plots for each stressed fraction analysed for a 7.14 mg mL^{-1} mAb solution.*

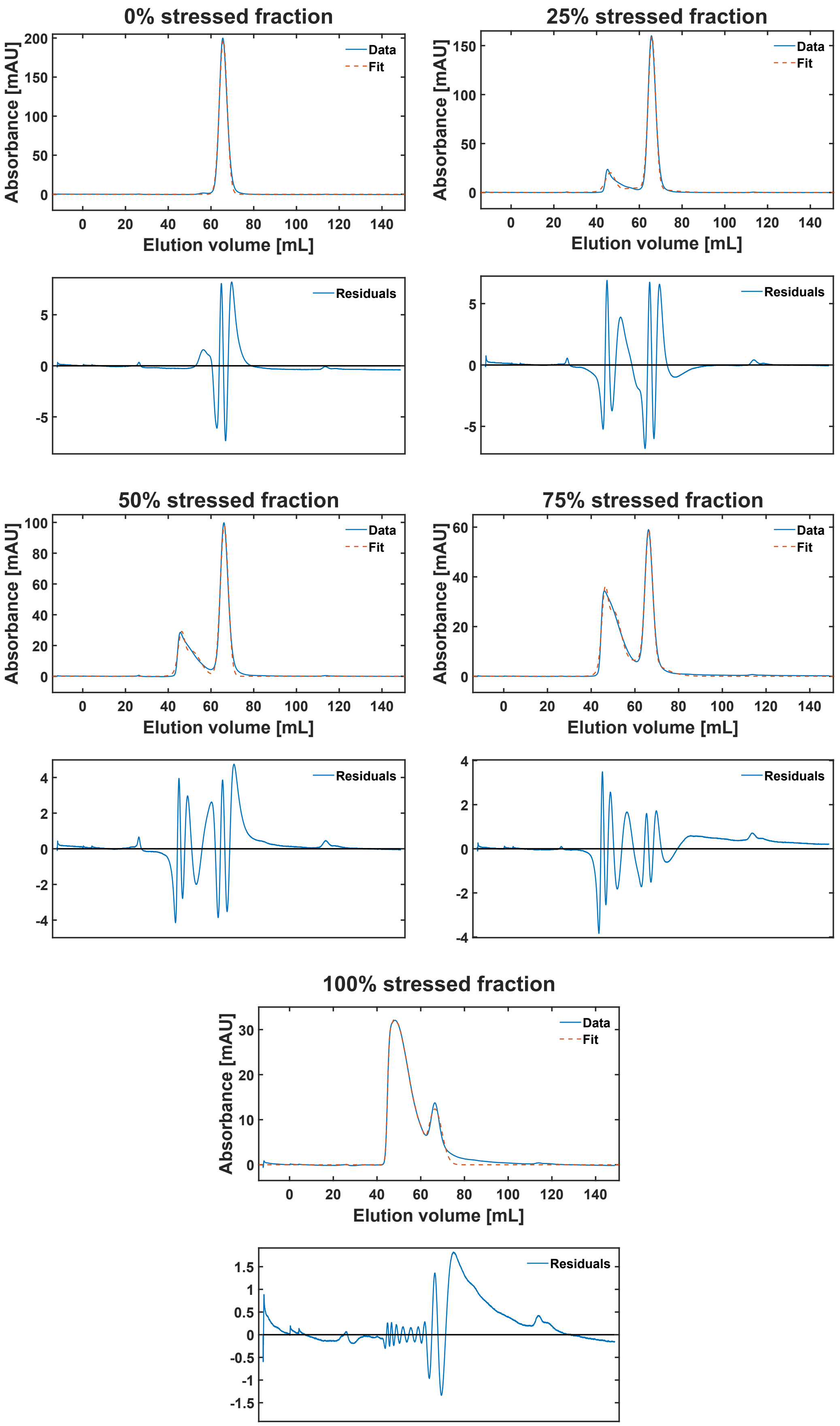

Figure S14 *– Fits and residuals plots for each stressed fraction analysed for a 9.81 mg mL*$^{-1}$ *mAb solution.*

Example NMR data

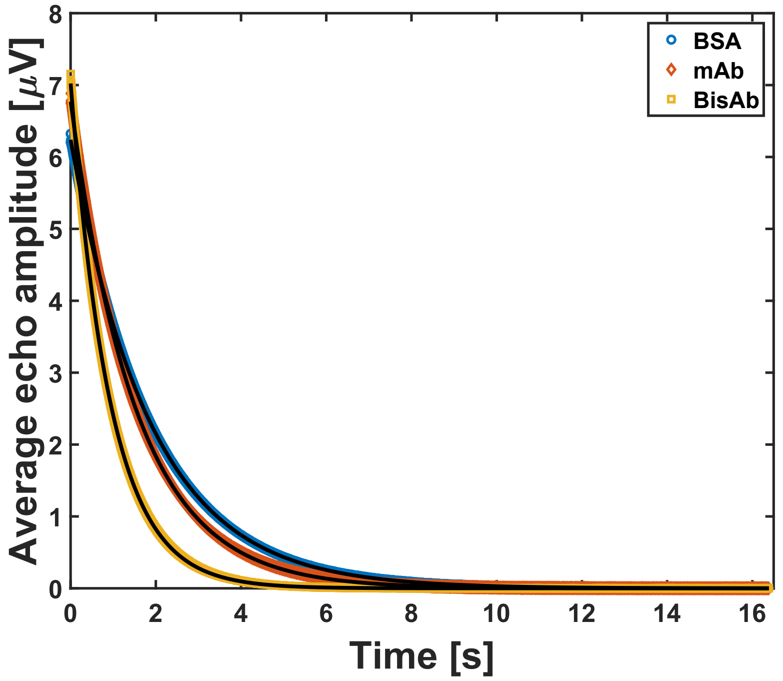

Figure S15 *– Example data recorded during a "T2Bulk" "one-shot" Carr-Purcell-Meiboom-Gill (CPMG) experiment for BSA (blue circles), mAb (orange diamonds) and BisAb (yellow squares). The associated fits (black lines) for each were determined in MATLAB 2022a (Mathworks, US).*

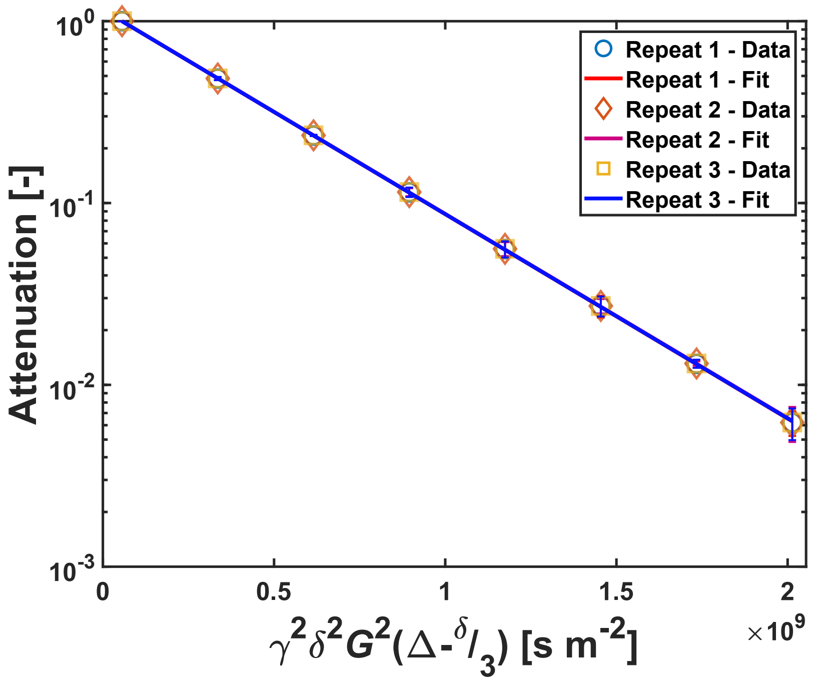

Figure S16 *– Example data obtained during a "PGSE" pulsed gradient spin echo experiment for three repeat measurements on a 9.62 mg mL^{-1}, 0% stressed fraction BSA solution. The associated fits and residual for each datapoint were determined in MATLAB 2022a (Mathworks, US). The residuals are multiplied by a factor of 10 for visibility.*

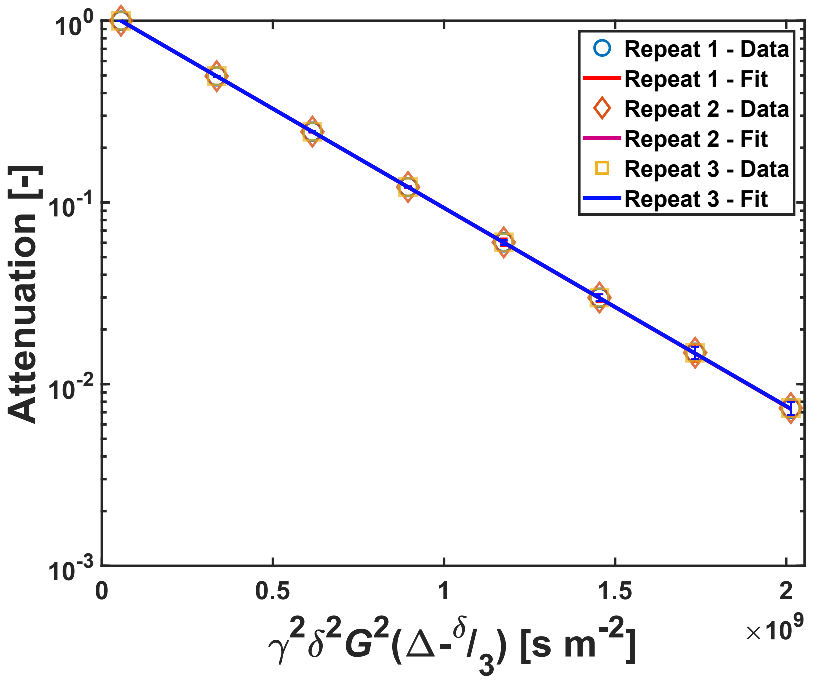

Figure S17 *– Example data obtained during a "PGSE" pulsed gradient spin echo experiment for three repeat measurements on a 9.81 mg mL^{-1}, 0% stressed fraction mAb solution. The associated fits and residuals for each datapoint were determined in MATLAB 2022a (Mathworks, US). The residuals are multiplied by a factor of 10 for visibility.*

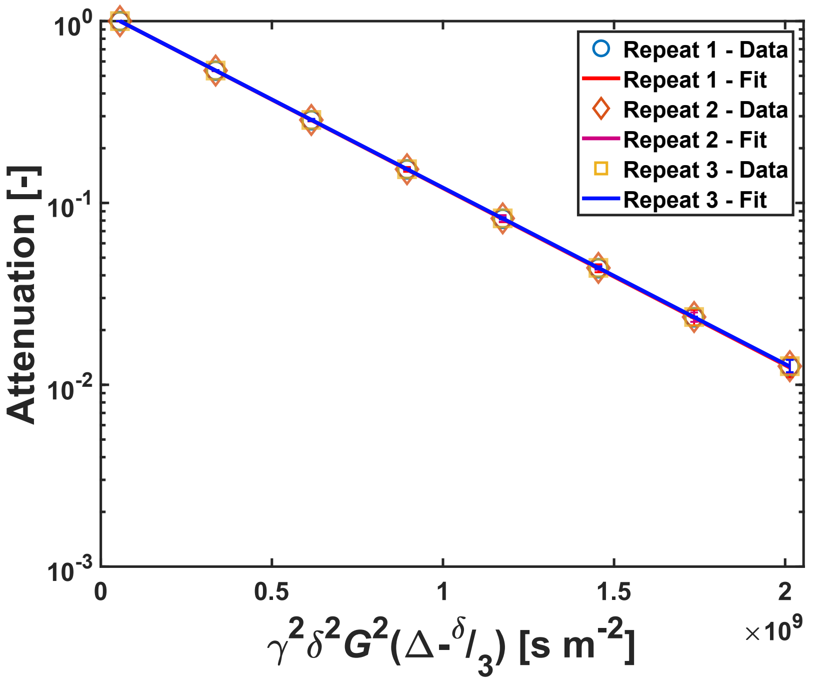

Figure S18 *– Example data obtained during a "PGSE" pulsed gradient spin echo experiment for three repeat measurements on a 9.03 mg mL^{-1} BisAb solution before heat-stress. The associated fits and residuals for each datapoint were determined in MATLAB 2022a (Mathworks, US). The residuals are multiplied by a factor of 10 for visibility.*

Dynamic light scattering analysis

Concentration of mAb solution used – 10.07 ± 0.01 mg mL^{-1}

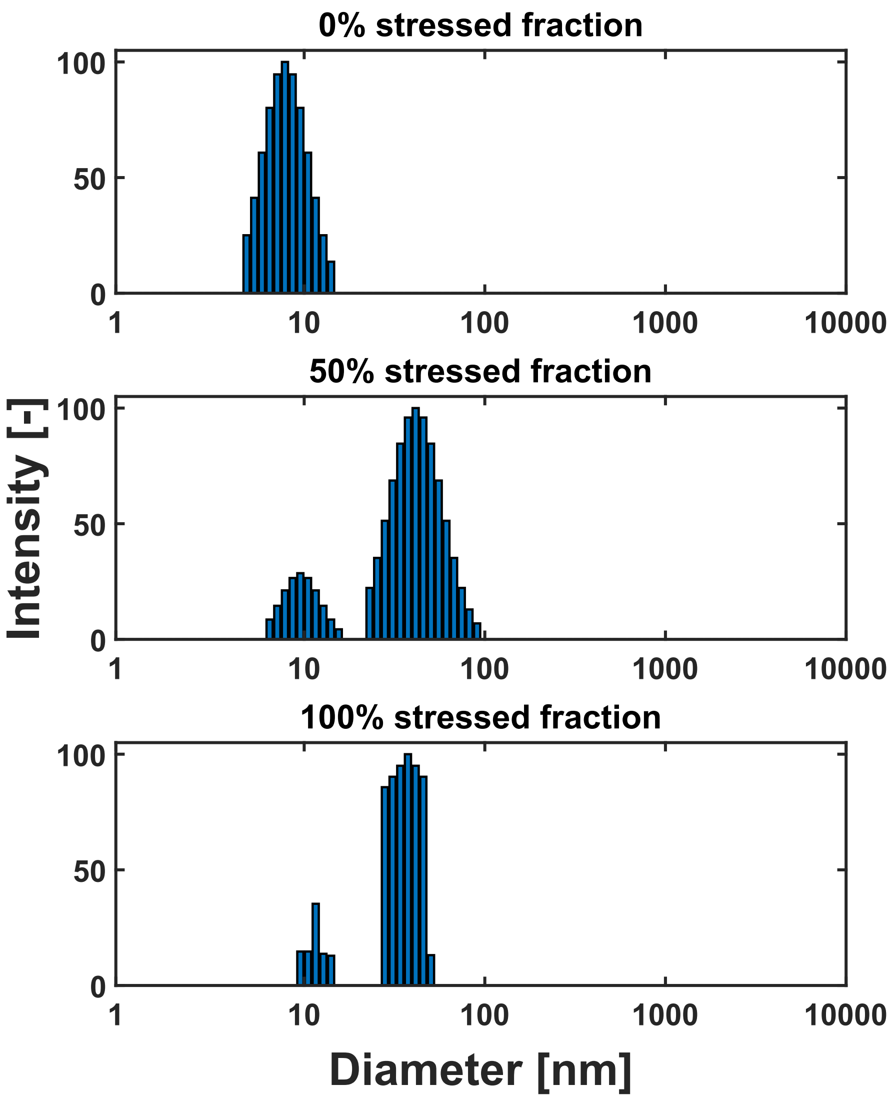

Figure S19 *– Example size distributions recorded by dynamic light scattering analysis for a 10.07 mg mL^{-1} mAb solution at different stressed fractions (0, 50, 100%).*

Table S4 *– Average effective diameter recorded for a 10.07 mg mL^{-1} mAb solution at different stressed fractions.*

Stressed fraction / %	***Average effective diameter / nm***	***Standard deviation***
0	8.1	± 0.06
50	31.8	± 0.78
100	26.9	± 0.12

The behaviour of $R_1(^1H_2O)$ with increased stressed fraction for BSA solutions

To determine the water longitudinal relaxation time [$T_1(^1H_2O)$], the "T1" pulse sequence was used. This is provided by the manufacturer and used without modification. The repetition time was kept at 10 s, with 4 scans accumulated. The "T1" pulse sequence is an inversion recovery sequence.[1] To obtain the longitudinal relaxation rate, the longitudinal relaxation time (T_1) for the water signal is first extracted by fitting the data to Equation S1:

$$I_t = I_0\left(1 - 2\exp\left(-\frac{t}{T_1}\right)\right) \quad \text{(S1)}$$

where I_t is the signal intensity at time t, and I_0 is the signal intensity at t = 0. A single component fit was used to give $T_1(^1H_2O)$, the reciprocal of which gives $R_1(^1H_2O)$. In these experiments, the following parameters were used: 20 μs radiofrequency (RF) pulse, with a 90° of -6 dB; 200 μs dwell time; 16384 points; 16 delay steps, spaced linearly between a minimum value of 1 ms and a maximum value of 15 s. The total experiment time for one measurement was approximately 14 min.

All NMR data was processed, analysed and visualised in MATLAB R2020b or R2022a (MathWorks, US), using custom scripts. All measurements were recorded in triplicate, with the arithmetic mean value taken to give the sample value; the standard error of this value was used to determine sample error. As the data shown in Figures S20 and S21 has been included for reference only, actual sample values and associated errors are not given.

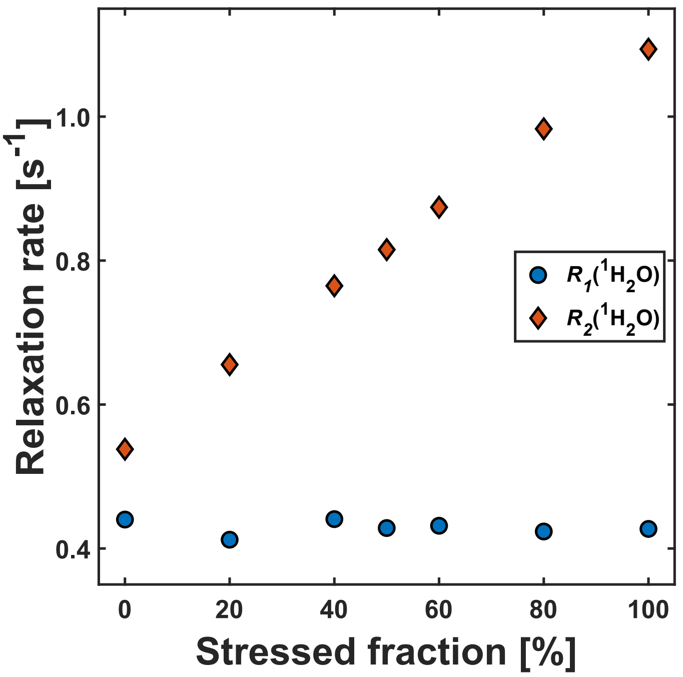

Figure S20 *– Plot showing the effect of stressed fraction on $R_1(^1H_2O)$ [circles] and $R_2(^1H_2O)$ [diamonds] for a BSA solution with a nominal concentration of 12.5 mg mL^{-1} (unverified). Sample errors were determined by taking the standard error of the arithmetic mean of three sample measurements. Error bars are excluded for clarity where the errors are smaller than the symbols used.*

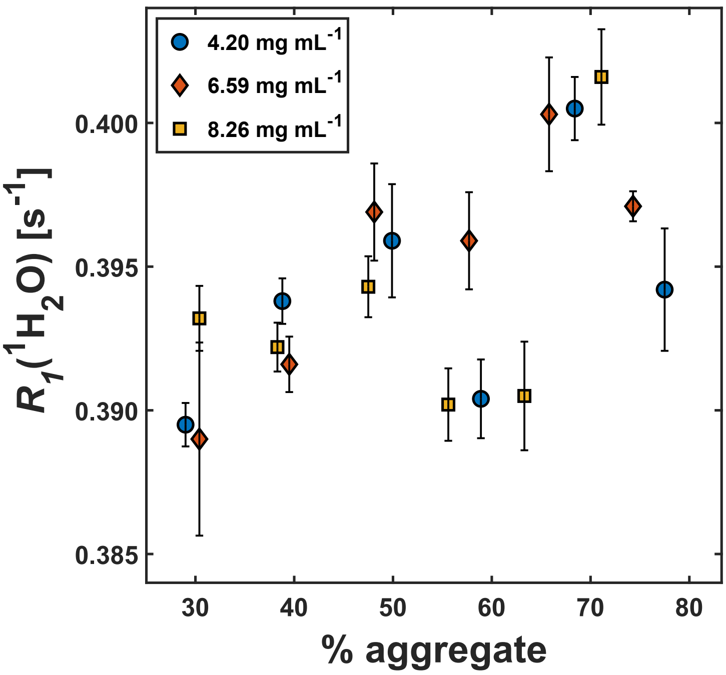

Figure S21 *– Plot showing the effect of aggregate percentage on $R_1(^1H_2O)$ for different BSA concentrations (4.20 mg mL^{-1} – circles; 6.59 mg mL^{-1} – diamonds; 8.26 mg mL^{-1} – squares). Sample errors were determined by taking the standard error of the arithmetic mean of three sample measurements.*

Data – BisAb

Data table

Table S5 *– Data for each BisAb solution studied in this work, before (Pre-HT) and after (Post-HT) heat-stress. The water transverse relaxation rate [$R_2(^1H_2O)$] and water diffusion coefficient [$D(^1H_2O)$] were determined by NMR analysis. Sample errors were determined by taking the standard error of the arithmetic mean of three sample measurements.*

Concentration / mg mL^{-1}		***$R_2(^1H_2O)$ / s^{-1}***	***$D(^1H_2O)$ / × 10^{-9} m^2 s^{-1}***
1.92	Pre-HT	0.5079 ± 0.0002	2.5654 ± 0.0015
	Post-HT	0.5052 ± 0.0003	2.5758 ± 0.0025
3.60	Pre-HT	0.6496 ± 0.0002	2.4882 ± 0.0010
	Post-HT	0.6909 ± 0.0003	2.4884 ± 0.0005
5.26	Pre-HT	0.7924 ± 0.0003	2.4090 ± 0.0009
	Post-HT	0.8990 ± 0.0002	2.4042 ± 0.0014
7.04	Pre-HT	0.9388 ± 0.0003	2.3143 ± 0.0015
	Post-HT	1.1383 ± 0.0007	2.3137 ± 0.0014
9.03	Pre-HT	1.0714 ± 0.0007	2.2370 ± 0.0021
	Post-HT	1.4105 ± 0.0007	2.2266 ± 0.0013

Size-exclusion chromatogram

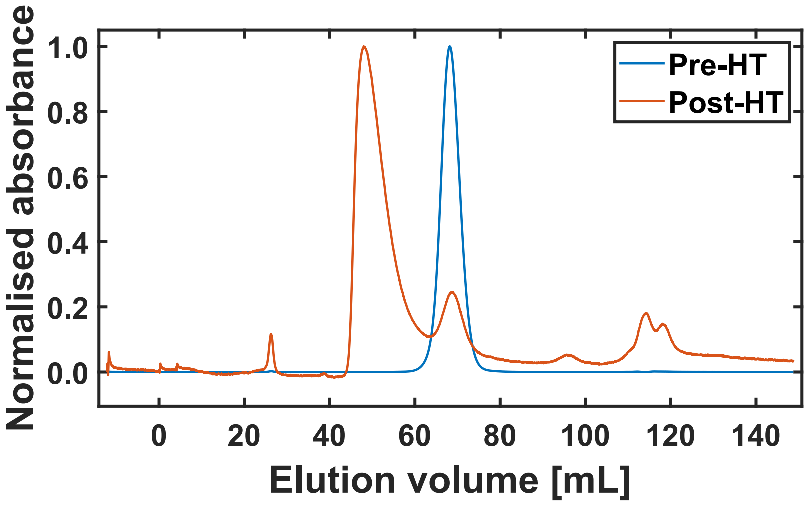

Figure S22 *– Normalised SEC chromatogram of a 9.03 mg mL^{-1} BisAb solution, before (Pre-HT) and after (Post-HT) heat-stress. The peak at an elution volume of approximately 25 mL is a column artefact; the peak at approximately 120 mL is histidine, a non-protein component of the BisAb concentrate used.*

Method flowchart

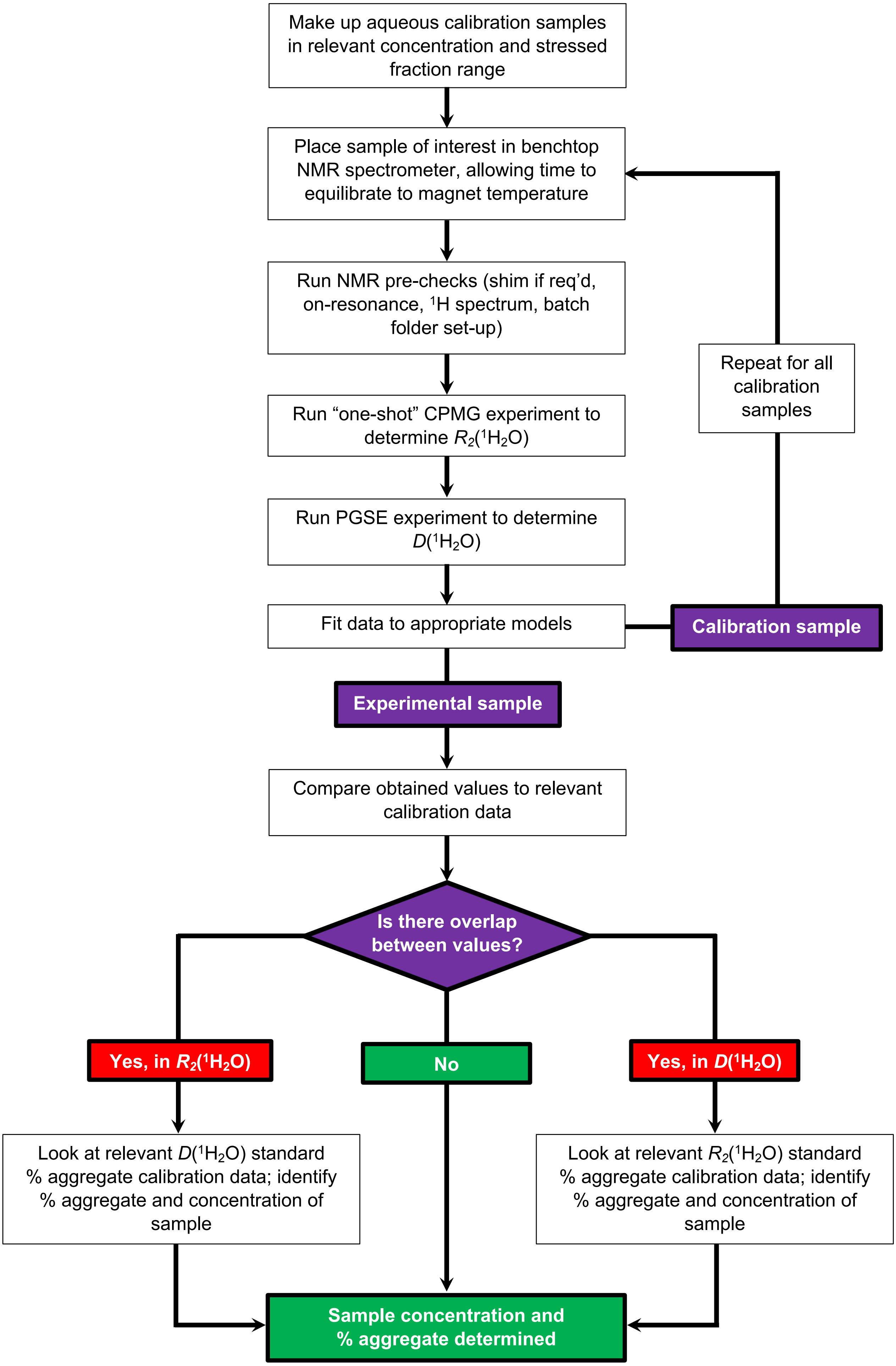

Case studies

First case study – BSA

A sample of BSA was studied by a user, and a $R_2(^1H_2O)$ value of 0.555 s^{-1}, and a $D(^1H_2O)$ value of 2.586 × 10^{-9} m^2 s^{-1}, were calculated.

The standard calibration data for $D(^1H_2O)$ and $R_2(^1H_2O)$ is consulted (Figure S23):

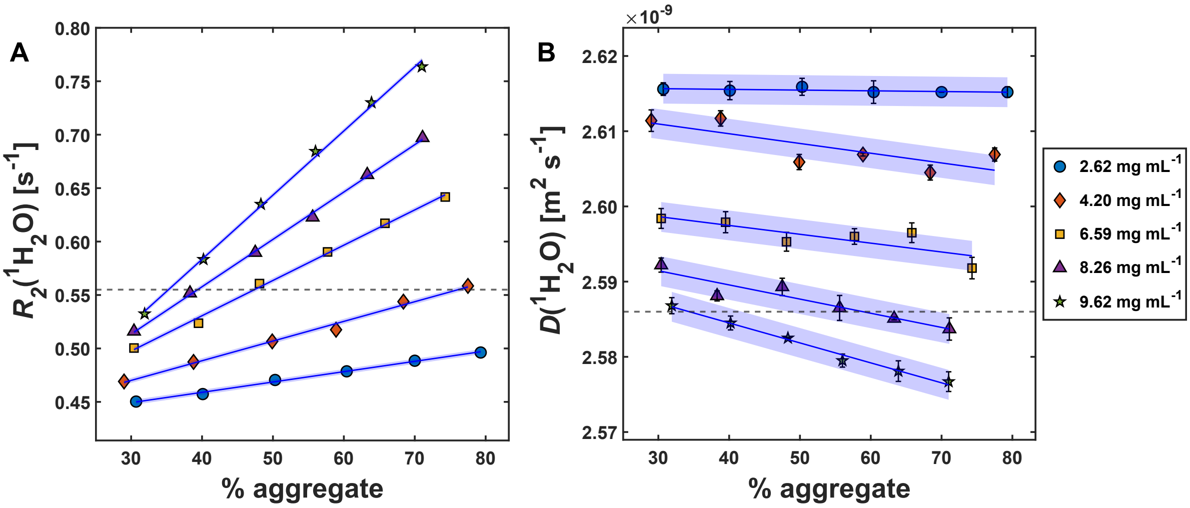

Figure S23 *– Plots showing the effect of concentration and aggregate percentage for known BSA calibration samples with $R_2(^1H_2O)$ (A) and $D(^1H_2O)$ (B). The dashed line in (A) represents a constant $R_2(^1H_2O)$ value of 0.555 s^{-1}. The dashed line in (B) represents a constant $D(^1H_2O)$ value of 2.586 × 10^{-9} m^2 s^{-1}. The solid lines represent linear regression fits to individual sample concentrations and the blue shaded areas represent 95% confidence intervals calculated from average of the standard error of all data points. In (A), the 95% confidence intervals are multiplied by a factor of 5 for visibility. Sample errors were determined by taking the standard error of the arithmetic mean of three sample measurements; values can be found in Table S2. Error bars are excluded for clarity where the errors are smaller than the symbols used.*

The $D(^1H_2O)$ value of the unknown sample is identified on the *y*-axis of the reference data plot, and a horizontal line is drawn (see Figure S23B).

A MATLAB script was written to create a (5 × 10) matrix of upper and lower limits of the 95% confidence intervals (obtained from the linear regression fits to the data shown in Figure S23B) for each different BSA sample concentration shown in Figure S23B; this is called "*ci_array*". The full MATLAB code can be found on Page S24 of the Supporting Information. A (5 × 10) matrix of identical $D(^1H_2O)$ values (*e.g.* 2.586 × 10^{-9} m^2 s^{-1} in this example) was then created; this is called "*d_array*". The following logical comparison was then performed for the two matrices to identify any overlap of *D* values within 0.1% of the upper and lower limts of "*ci_array*", thus yielding a Boolean array which we call "*truth_array*":

```
truth_array = abs(ci_array - d_array) <= threshold*abs(ci_array)
```

Using this logic, the unknown BSA sample with $D(^1H_2O)$ = 2.586 × 10^{-9} m^2 s^{-1} intercepts the 95% confidence interval array at only two different concentrations, but at a number of different % aggregate levels. Figure S24 represents the truth array generated:

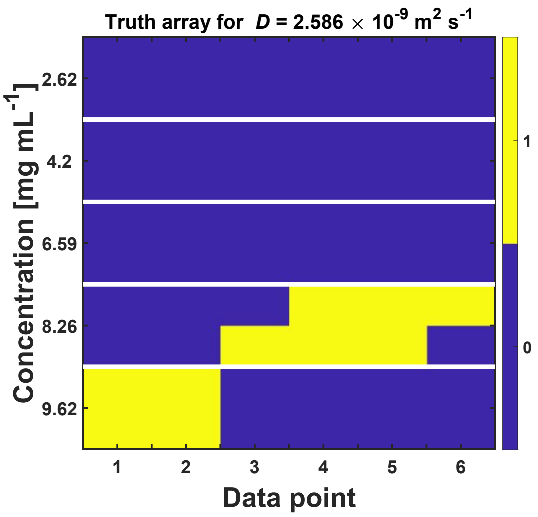

Figure S24 *– Truth array generated using standard calibration data for a BSA sample with a D(^1H_2O) value of 2.586 × 10^{-9} m^2 s^{-1}. Yellow represents a true value, while blue represents a false value.*

From this truth array, the concentrations and % aggregate shows that the sample could be any one of the following identities:

(D_i) *9.62 mg mL^{-1}, ~32% aggregate.*
(D_{ii}) *9.62 mg mL^{-1}, ~40% aggregate.*
(D_{iii}) *8.26 mg mL^{-1}, ~48% aggregate.*
(D_{iv}) *8.26 mg mL^{-1}, ~56% aggregate.*
(D_v) *8.26 mg mL^{-1}, ~64% aggregate.*
(D_{vi}) *8.26 mg mL^{-1}, ~71% aggregate.*

A horizontal line is then drawn on the reference R_2(^1H_2O) data at the obtained value of 0.555 s^{-1} (see Figure S23A). It is seen that this line intercepts the 95% confidence intervals of the R_2(^1H_2O) reference data at four different (and unique) BSA concentrations:

($R_{2,i}$) *9.62 mg mL^{-1}, ~36% aggregate.*
($R_{2,ii}$) *8.26 mg mL^{-1}, ~38% aggregate.*
($R_{2,iii}$) *6.59 mg mL^{-1}, ~48% aggregate.*
($R_{2,iv}$) *4.59 mg mL^{-1}, ~78% aggregate.*

The only assignment for the unknown BSA sample is therefore a concentration of 9.62 mg mL^{-1}, with 32-36% aggregate.

Second case study – mAb

A sample of mAb was studied by a user, and a $R_2(^1H_2O)$ value of 0.590 s^{-1}, and a $D(^1H_2O)$ value of 2.5550 × 10^{-9} m^2 s^{-1}, were recorded.

The standard calibration data for $D(^1H_2O)$ and $R_2(^1H_2O)$ is consulted (Figure S25):

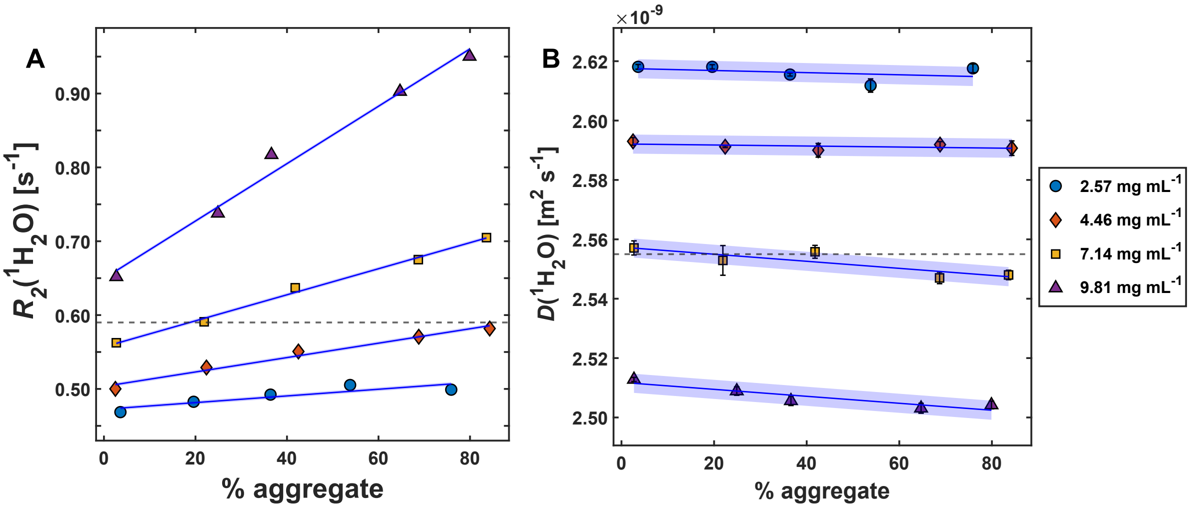

Figure S25 *– Plots showing the effect of concentration and aggregate percentage for known mAb calibration samples with $R_2(^1H_2O)$ (A) and $D(^1H_2O)$ (B). The dashed line in (A) represents a constant $R_2(^1H_2O)$ value of 0.590 s^{-1}. The dashed line in (B) represents a constant $D(^1H_2O)$ value of 2.555 × 10^{-9} m^2 s^{-1}. The solid lines represent linear regression fits to individual sample concentrations and the blue shaded areas represent 95% confidence intervals calculated from average of the standard error of all data points. In (A), the 95% confidence intervals are multiplied by a factor of 5 for visibility. Sample errors were determined by taking the standard error of the arithmetic mean of three sample measurements; values can be found in Table S3. Error bars are excluded for clarity where the errors are smaller than the symbols used.*

The $R_2(^1H_2O)$ value of the unknown sample is identified on the *y*-axis of the reference data plot, and a horizontal line is drawn (see Figure S25A). Upon visual inspection, it is clear that the sample could have two potential identities:

($R_{2,i}$)   4.46 mg mL^{-1}, ~85% aggregate.
($R_{2,ii}$)   7.14 mg mL^{-1}, ~ 22% aggregate.

A horizontal line is then drawn on the reference $D(^1H_2O)$ data at the obtained value of 2.555 × 10^{-9} m^2 s^{-1} (see Figure S25B). Upon visual inspection, it is seen that this line intercepts the 95% confidence intervals of the $D(^1H_2O)$ reference data at one mAb concentration:

(D_i)   7.14 mg mL^{-1}, ~42% aggregate.
(D_{ii})   7.14 mg mL^{-1}, ~69% aggregate.
(D_{iii})   7.14 mg mL^{-1}, ~84% aggregate.

Based on the obtained $D(^1H_2O)$ value, the user can determine that the concentration of the sample is 7.14 mg mL^{-1}.

The only assignment for the unknown mAb sample is therefore a concentration of 7.14 mg mL^{-1}, with ~22% aggregate.

Comparison of 0% and 100% SF data for BSA and mAb

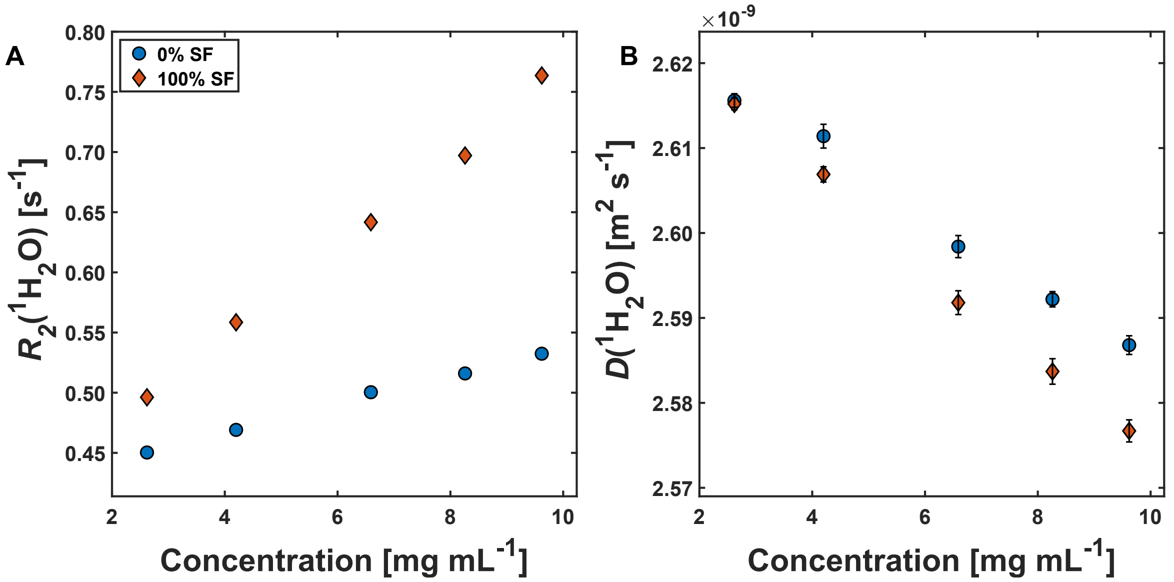

Figure S26 *– Plots showing the effect of concentration with $R_2(^1H_2O)$ (A) and $D(^1H_2O)$ (B) for BSA solutions with 0% (circles) and 100% (diamonds) stressed fraction. Sample errors were determined by taking the standard error of the arithmetic mean of three sample measurements; values can be found in Table S2. Error bars are excluded for clarity where the errors are smaller than the symbols used.*

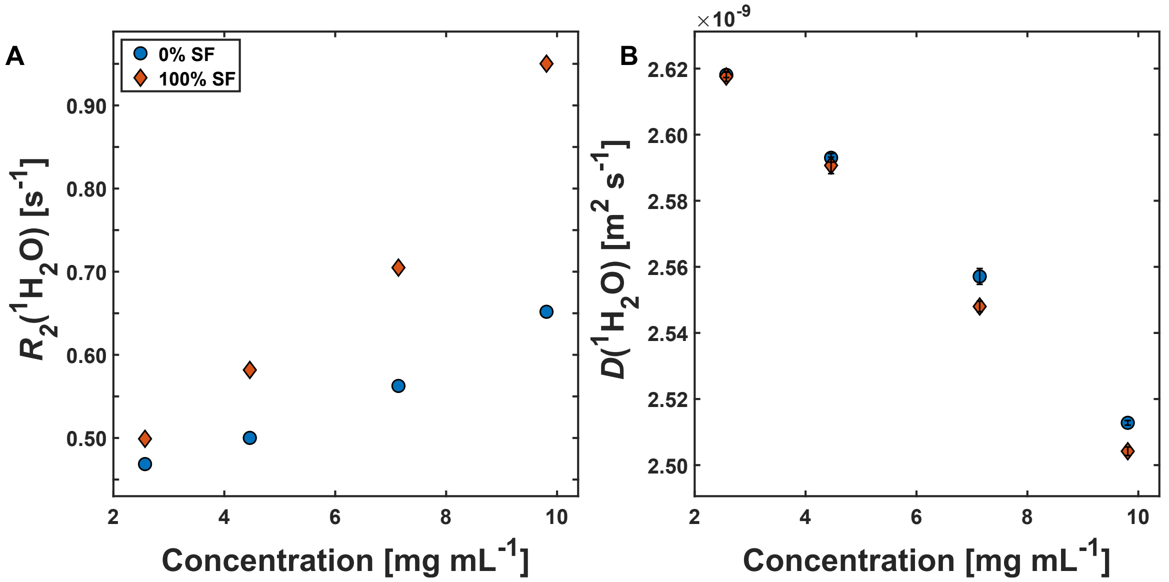

Figure S27 *– Plots showing the effect of concentration with $R_2(^1H_2O)$ (A) and $D(^1H_2O)$ (B) for mAb solutions with 0% (circles) and 100% (diamonds) stressed fraction. Sample errors were determined by taking the standard error of the arithmetic mean of three sample measurements; values can be found in Table S3. Error bars are excluded for clarity where the errors are smaller than the symbols used.*

MATLAB code to generate truth array

```
%% Generate lin fits with 95% confidence interval and truth array
%
% Written by Mark I. Grimes and Mick D. Mantle, University of Cambridge, March
2024.
%
% Data should be inputted as an (n x [m*2]) matrix called "all_data", where n is
the number of concentrations, and m is the number of stressed fractions studied.
% Data should be put in positions (:,1:m); errors should be put in positions
(:,m:end), following same order as data.
%
% Aggregate content values should be inputted as an (n x m) matrix called
"AggVals", where n is the number of concentrations, and m is the number of
stressed
% fractions studied.
%

critical_value = 1.96; % change as req'd; chosen for 95% confidence interval
generation

xRows = 1;
disp(' ');
xColumns = input('Please input the number of concentrations you have: '); % User
to input concentrations studied
disp(' ');

disp('Input concentration values: ');
Concs = zeros(xRows,xColumns);
for k = 1:xRows
    for m = 1:xColumns
        Concs(k,m) = input("Input the matrix value for (" + k + "," + m +"): ");
    end
end

f1 = figure;
count1 = 0;
y_pred=[];x_pred=[];
Markers = {'ok','dk','sk','^k','pk'}; % for marker style - change as req'd
MarkerFC = {[0 0.4470 0.7410],[0.8500 0.3250 0.0980],[0.9290 0.6940
0.1250],[0.4940 0.1840 0.5560],[0.4660 0.6740 0.1880]}; % for marker colours -
change as req'd

for j = 1:size(all_data,1)

x = AggVals(j,:);

% Scatter plot
DataPlots(j) = plot(x, all_data(1 + count1, 1:6), Markers{j}, 'MarkerSize',
12,'LineWidth',1.5,'MarkerFaceColor',MarkerFC{j});

hold on;

% Error bars
errorbar(x, all_data(1 + count1, 1:6), all_data(1 + count1, 7:end), 'k',
'LineStyle', 'None', 'CapSize', 5,'linewidth',1.5);

% Linear regression
coefficients = polyfit(x, all_data(1 + count1, 1:6), 1);
line = polyval(coefficients, x);
plot(x, line, 'Color', 'b', 'LineWidth',1.5);
```

```
% Confidence intervals
x_pred = x;
y_pred(j,:) = polyval(coefficients, x_pred);

result = mean(all_data(:,7:end), 'all');
result_array = zeros(6) + result;

y_err = result_array;  % Assuming the error bars represent the standard error of
the mean
y_pred_upper = y_pred(j,:) + critical_value * y_err(j); % Generate values for
confidence interval fill area
y_pred_lower = y_pred(j,:) - critical_value * y_err(j);

% Fill between confidence intervals
fill([x_pred, fliplr(x_pred)], [y_pred_lower, fliplr(y_pred_upper)], 'b',
'FaceAlpha', 0.2, 'EdgeColor', 'none');


count1 = count1 + 1;
end

hold off;

% Plot data
legend([DataPlots],num2str(Concs','%5.2f mg mL^{-1}'),'Location','EastOutside'); %
change unit as req'd
set(gca,'FontSize',20,'FontWeight','bold','LineWidth',2,'box','on');
xlabel('% aggregate','FontSize',30)
ylabel('{\it D}(^1H_2O) [m^2 s^{-1}]', 'FontSize',30)
f1.Position = [400 50 1200 750];
set(gcf, 'renderer', 'painters')
ylim([(min(min(all_data(:,1:6)))*0.997) (max(max(all_data(:,1:6)))*1.003)]) % set
as req'd
xLimBuffer = max(max(AggVals))*1.05 - max(max(AggVals));
xlim([(min(min(AggVals)))-xLimBuffer max(max(AggVals))+xLimBuffer])
yticks([2.57e-9 2.58e-9 2.59e-9 2.6e-9 2.61e-9 2.62e-9]) % set as req'd
axis square
ytickformat('%.2f')

%% Set up +/- confidence intervals array will be (2*m x n) in size
% Also set up +/- D values array of same dimensions to see when values are equal

D = input("Input the diffusion value of interest: ");  % User to input diffusion
coefficient value of interest
D_array = zeros(1,6) + D;  % make a 1D array of 6 identical values

result = mean(all_data(:, 7:end), 'all');  % calculates average of errors
result_array = zeros(1,6) + result;  % repeats single value 6 times

D_upper = D_array + result_array;  % upper value of D array
D_lower = D_array - result_array;  % lower value of D array

y_pred_L = [];  % create dummy cell array to store upper 95% conf
y_pred_U = [];  % create dummy cell array to store lower 95% conf

y_err2 = result_array;  % set y_error to result array (is a single value)
ci_array = zeros(10, 6);
d_array = zeros(10, 6);
count1 = 1;
y_pred_upper2 = y_pred + (critical_value * y_err2);
y_pred_lower2 = y_pred - (critical_value * y_err2);

for j = 1:2:(size(all_data,1)*2)
```

```
    y_pred_U = y_pred_upper2(count1,:);  % stores this upper value for one BSA
conc
    ci_array(j,:) = y_pred_U;
    d_array(j,:) = D_upper;

    count1 = count1 + 1;
end

count1 = 1;
for k = 2:2:(size(all_data,1)*2)
    y_pred_L = y_pred_lower2(count1,:);  % stores this Lower value for one BSA
conc
    ci_array(k,:) = y_pred_L;
    d_array(k,:) = D_lower;
    count1 = count1 + 1;
end

% Set the threshold for comparison (0.1%)
threshold = 0.001;

% Perform element-wise comparison
truth_array = abs(ci_array - d_array) <= threshold*abs(ci_array);

% Display the comparison array in command window
disp('Comparison Array:');
disp(truth_array);

% Plot the comparison array
f2 = figure;
y_ticks = 1.5:2:9.5; % change as req'd
x_ticks = 0.5:0.5:6; % change as req'd
y_labels = Concs;
x_labels = [' ';'1';' ';'2';' ';'3';' ';'4';' ';'5';' ';'6']; % change as req'd
imagesc(truth_array);
hold on
yline(2.5,'Color','w','LineWidth',5,'Alpha',1);
yline(4.5,'Color','w','LineWidth',5,'Alpha',1) % lines for formatting; change
number if req'd
yline(6.5,'Color','w','LineWidth',5,'Alpha',1);
yline(8.5,'Color','w','LineWidth',5,'Alpha',1)
title("Truth array for {\it D} = " + (D*10^9) + " \times 10^{-9} m^2 s^{-1}");
set(gca, 'XTick', x_ticks, 'XTickLabel', x_labels, 'YTick',
y_ticks,'YTickLabel',y_labels);
set(gca,'FontSize',20,'FontWeight','bold','LineWidth',2,'box','on');
ylabel('Concentration [mg mL^{-1}]', 'FontSize',30)
f2.Position = [400 50 950 750];
set(gcf, 'renderer', 'painters')
axis square
colormap(parula(2));
clb = colorbar;
clb.Ticks = [0.25 0.75];
clb.TickLabels = [0 1];
```

References

(1) Hahn, E. L. An Accurate Nuclear Magnetic Resonance Method for Measuring Spin-Lattice Relaxation Times. *Phys. Rev.* **1949**, *76* (1), 145–146. https://doi.org/10.1103/PhysRev.76.145.
